# High-throughput genome sequencing of lichenizing fungi to assess gene loss in the ammonium transporter/ammonia permease gene family

**DOI:** 10.1186/1471-2164-14-225

**Published:** 2013-04-04

**Authors:** Tami R McDonald, Olaf Mueller, Fred S Dietrich, François Lutzoni

**Affiliations:** 1 Department of Biology, Duke University, Durham, NC 27708, USA; 2 Department of Molecular Genetics and Microbiology, Duke University, Durham, NC 27710, USA; 3 Current address: Department of Microbiology, University of Minnesota, Minneapolis, MN 55455, USA

**Keywords:** Ammonium transporter, Ammonia permease, Phylogeny, Lichen symbiosis, Fungi, Genome, Illumina, Horizontal gene transfer

## Abstract

**Background:**

Horizontal gene transfer has shaped the evolution of the ammonium transporter/ammonia permease gene family. Horizontal transfers of ammonium transporter/ammonia permease genes into the fungi include one transfer from archaea to the filamentous ascomycetes associated with the adaptive radiation of the leotiomyceta. The horizontally transferred gene has subsequently been lost in most of the group but has been selectively retained in lichenizing fungi. However, some groups of lichens appear to have secondarily lost the archaeal ammonium transporter. Definitive assessment of gene loss can only be made via whole genome sequencing.

**Results:**

Ammonium transporter/ammonia permease gene sequences were recovered from the assembled genomes of eight lichenizing fungi in key clades including the Caliciales, the Peltigerales, the Ostropomycetidae, the Acarosporomycetidae, the Verrucariales, the Arthoniomycetidae and the Lichinales. The genes recovered were included in a refined phylogenetic analysis. The hypothesis that lichens symbiotic with a nitrogen-fixing cyanobacterium as a primary photobiont or lichens living in high nitrogen environments lose the plant-like ammonium transporters was upheld, but did not account for additional losses of ammonium transporters/ammonia permeases in the lichens from the Acarosporomycetidae, Chaetotheriomycetes and Arthoniomycetes. In addition, the four ammonium transporter/ammonia permease genes from *Cladonia grayi* were shown to be functional by expressing the lichen genes in a strain of *Saccharomyces cerevisiae* in which all three native ammonium transporters were deleted, and assaying for growth on limiting ammonia as a sole nitrogen source.

**Conclusions:**

Given sufficient coverage, next-generation sequencing technology can definitively address the loss of a gene in a genome when using environmental DNA isolated from lichen thalli collected from their natural habitats. Lichen-forming fungi have been losing ammonium transporters/ammonia permease genes at a slower rate than the most closely related non-lichenized lineages. These horizontally transferred genes in the *Cladonia grayi* genome encode functional ammonium transporters/ammonia permeases.

## Background

Lichens are symbioses between a fungus and a photosynthesizing partner such as a green alga, a cyanobacterium, or both. About one-fifth of all known fungi lichenize, and for these fungi the symbiosis is, with few exceptions, obligate. Unlike mycorrhizal or rhizobial symbioses, lichen symbioses are not well understood. This is mainly because lichens grow very slowly (≤1 cm a year), it is very difficult to grow the fungus and the alga separately and, moreover, it is not yet possible to resynthesize the mature symbiosis in the laboratory from the fungal and photobiont partners isolated in axenic culture [1-4]. Also, it is not yet possible to delete genes, nor has any transformation method been established to introduce genes into the genomes of any lichenizing fungus or alga. However, the lack of genetic tools for these intractable organisms has been partially compensated for by the advent of high-throughput genome sequencing.

Previously, the genomes of the model lichen *Cladonia grayi*, made up of the lichenizing fungus *Cladonia grayi* for which the association is named, and its green algal partner *Asterochloris* sp., were sequenced at Duke University [5,6]. We searched the genome sequences for evidence of horizontal gene transfer [7-9] between the lichen symbionts; that is, whether there were genes of algal origin in the fungal genome or genes of fungal origin in the algal genome. A thorough homology search of all the genes in each genome revealed that two genes in the fungal genome appeared to have been horizontally transferred, although not from green algae. Both genes encoded ammonium transporters [10].

Ammonium transporters/ammonia permeases (AMTPs) are highly conserved proteins found in most organisms, including prokaryotes and eukaryotes. These proteins are composed of 11 transmembrane helices that fold to form a pore through which ammonia or ammonium moves [11,12]. In their native conformation they trimerize, forming a tripartite pore [13]. While some AMTPs have been shown to transport ammonium (NH_4_^+^), notably those proteins in the AMT2 family of land plants [14,15], most AMTPs have been shown to transport ammonia (NH_3_) [16-22]. Proteins in the related Rh family [23] have 12 transmembrane domains and have been shown to conduct ammonia and in some cases CO_2_[24-26].

The evolutionary history of this family of genes is complex, involving lineage-specific gene family expansions, contractions, and losses as well as ancient and recent horizontal gene transfer events. Fungal AMTPs are in a phylogenetic clade by themselves that includes both low-affinity [27-30] and high-affinity [31-35] AMTPs (MEP γ clade [10]). The history of these genes in the fungi is particularly complicated, appearing to commence with an ancient horizontal gene transfer event of high-affinity AMTPs of prokaryotic origin during the early evolution of the fungi, followed by several lineage-specific gene-family expansions, as well as a duplication and neofunctionalization event in the early evolution of the Dikarya that lead to the evolution of low-affinity AMTPs [10].

In addition to these events, a second horizontal gene transfer event of high-affinity AMTPs occurred in the early evolution of filamentous ascomycetes (associated with a putative adaptive radiation of the leotiomyceta [10,36,37]). These horizontally transferred AMTPs are distinct from the fungal high- and low-affinity AMTPs of the MEP γ clade, and in fact are most closely related to AMTPs from land plants in the AMT2 family e.g. [12,15,35,38-41] and to transporters from mostly hyperacidophilic chemoautolithotrophic prokaryotes inhabiting deep sea thermal vents [42], volcanic hotsprings and thermal vents [43-48], acid mine drainages [49-55], and similar extreme environments (MEP α clade) [10].

Interestingly, only a subset of the leotiomyceta, most of which are symbiotic with green algae in lichen symbioses, have representatives of this new clade of AMTPs in their genomes. In fact, lichenizing fungi in three different taxonomic classes of fungi, including the Lecanoromycetes, the Eurotiomycetes and the Dothideomycetes [36,56,57], have actually duplicated these genes. By contrast, as of 2012 only four non-lichenizing fungi in two genera (*Penicillium* with *Talaromyces* teleomorphs, *Fusarium* with *Gibberella* teleomorphs) out of more than 200 publicly available sequenced fungal genomes have representatives of this new clade of AMTPs in their genomes, and these transporters are not duplicated. This result suggests that lichenized fungi have preferentially retained the MEP α gene after the initial horizontal gene transfer event during the early evolution of the filamentous ascomycetes, while non-lichenized fungi have lost this gene [10].

The MEP α gene was not found in all lichens surveyed. In particular, the MEP α gene was never recovered from the two orders of lichens most closely related to the order in which the original discovery was made. In one of these two orders, the Peltigerales [58], the lichens are symbiotic with nitrogen-fixing cyanobacteria. In the other order, the Teloschistales [59], many lichens inhabit high-nitrogen niches, like bird perching sites. The availability of nitrogen sources from the environment or from a symbiont, coupled with the failure to identify the AMTP gene by PCR suggests that lichens in these two orders may no longer need the MEP α AMTP and may have shed it from their genomes.

Here, we further characterize this new clade of fungal AMTPs. We sequence the genomes of eight lichenizing fungi in key lineages that may have shed the AMTPs and search the genomes for the horizontally-transferred AMTPs. We correlate the presence of the AMTPs of the new clade with nitrogen lifestyle by surveying lichen fungi that are closely related to the main lineages previously examined but that tolerate high-nitrogen habitats, or that employ nitrogen-fixing cyanobacteria rather than green algae as the primary symbionts. We also characterize the function of the AMTPs from one lichen, *Cladonia grayi*, by assaying for growth on ammonium as a sole nitrogen source. We present a phylogeny of fungal AMTPs to contextualize this clade.

## Results

### Genome assembly completeness

There is no way to definitively assess the absence of a gene from a genome except the sequencing of the whole genome. Even then, however, it could be argued that the gene is in fact present in the genome but is not found simply because the assembly is incomplete. In order to test completeness of the assemblies, the CEGMA (Core Eukaryotic Genes Mapping Approach) pipeline [60,61] was used. CEGMA queries against a core group of 248 highly conserved eukaryotic genes (CEGs). Completeness scores (Table 1) varied from 92-96% complete, and 97-98% partial detection of CEGs, respectively, for all genomes except *Physcia*. For comparison we assessed the two publicly available and well-assembled genomes of *Aspergillus fumigatus* AF293 [62] and *Neurospora crassa* or74a [63] with this method and determined scores of 96% complete and 97-98% partial CEGs detection. Data on the assemblies is shown in Table 2, and statistics on the raw data is presented in Table 3.

**Table 1:** Statistics on the completeness of lichen genome assemblies as determined by CEGMA

	***Acarospora strigata***	***Arthonia rubrocincta***	***Dibaeis baeomyces***^**M**^	***Endocarpon pallidulum***	***Graphis scripta***
Complete genes	94.35	93.95	95.16	94.76	95.97
Partial genes	98.39	97.58	97.18	97.18	98.15
	***Leptogium*austroamericanum**^**M**^	***Peltula cylindrica***^**M**^	***Physcia*cf.*stellaris***^**M**^	***Aspergillus fumigatus***	***Neurospora crassa***
Complete genes	94.76	92.74	73.39	96.37	95.56
Partial genes	97.58	97.98	92.34	97.98	97.18

^M^ = metagenome: Species names not followed by ^M^ denote genome projects from culture (all reads represent the lichenizing or free-living fungus); species names followed by ^M^ denote genome projects from lichens metagenomes (reads represent the lichenizing fungus, the lichenizing alga or cyanobacterium, and the associated microbial community).

**Table 2:** Statistics on the genome assemblies for five cultured lichens and metagenome assemblies for three lichen thalli

	***Acarospora strigata***	***Arthonia rubrocincta***	***Dibaeis baeomyces***^**M**^	***Endocarpon pallidulum***
size (Mb)	27	26	35	41
Scaffolds	938	550	1369	5508
N50 (kb)	81	102	70	40
	***Graphis scripta***	***Leptogium*austroamericanum**^**M**^	***Peltula cylindrica***^**M**^	***Physcia*cf*. stellaris***^**M**^
size (Mb)	36	46	32	59
Scaffolds	1453	9612	1885	24004
N50 (kb)	79	19	42	4

^M^ = metagenome.

**Table 3:** Statistics on the raw data for eight lichen genomes

	***Acarospora strigata***	***Arthonia rubrocincta***
Total sequences	80464902	103562964
first 30 bases high quality	36326870	44129871
first 50 bases high quality	31585425	36870361
first 70 bases high quality	27701126	30691804
all 75 bases high quality	26289676	28531448
Estimated high quality coverage	68×	82×
	***Dibaeis baeomyces***^**M**^	***Endocarpon pallidulum***
Total sequences	243144702	92018336
first 30 bases high quality	128915320	43595646
first 50 bases high quality	113229286	38937482
first 70 bases high quality	99892761	35132351
all 75 bases high quality	94764211	33697486
Estimated high quality coverage	80×	81×
	***Graphis scripta***	***Leptogium austroamericanum***^**M**^
Total sequences	92331310	204893442
first 30 bases high quality	42665651	93698059
first 50 bases high quality	37267191	77111438
first 70 bases high quality	32664052	66199646
all 75 bases high quality	30956142	62669378
Estimated high quality coverage	173×	148x
	***Peltula cylindrica***^**M**^	***Physcia cf. stellaris***^**M**^
Total sequences	152251382	201984964
first 30 bases high quality	79517806	90751190
first 50 bases high quality	67269367	78374611
first 70 bases high quality	57821683	68425794
all 75 bases high quality	28531448	64745297
Estimated high quality coverage	126×	57×

^M^ = metagenome. Estimated high quality coverage calculated by dividing the number of reads with at least the first 30 bases of high quality by the estimated genome size (of cultured fungi) or genome sizes (of lichenizing fungus and lichenizing alga or cyanobacterium but excluding the associated microbial community, and therefore overestimating coverage).

These data were of interest as of the eight lichens sequenced, four were actually metagenome projects. Lichens can be seen as micro-communities, hosting not just the mycobiont (fungal) and photobiont (algal or cyanobacterial symbionts), but a rich internal microbiota composed of bacteria and endolichenic fungi [64,65]. As lichenizing fungi have only infrequently been successfully cultured compared to other fungi (although see [66,67]), and because it can take up to three years to produce enough tissue to extract sufficient DNA for a genome sequence, sequencing a metagenome from thalli taken directly from nature would greatly reduce the time required for a genome project, were the final product of useful quality. The lichens used for metagenomes here included two cyanolichens symbiotic with cyanobacteria, and two lichens symbiotic with green algae, one of which has a very tight association with the algal symbiont, and one of which produces large fungal fruiting stalks (podetia) essentially devoid of the algal symbiont. The data show that where the symbiont is a cyanobacterium (prokaryote), or where the fungal portion of the thallus can be easily removed from the algal portion, the assembly suffers only a little compared to the assembly of a genome from a fungal culture. However, when the fungal and algal portions of the lichen cannot be easily dissected, as with *Physcia* cf. *stellaris*, the assembly is more problematic. It is therefore deemed not strictly necessary, but preferable, to sequence these fungi from culture where feasible.

In further test queries, 20 putative single-copy nuclear genes, which are conserved throughout the fungi were chosen as references. These genes are of approximately comparable size to the AMTP genes. If part or all of each of the 20 test sequences could be recovered from a genome, then it was considered likely that homology searches for genes of interest, i.e. AMTPs and particularly the MEP α gene(s), would identify at least a portion of the gene(s) if such were present in the genome. All 20 of the test genes were recovered from each of the eight genomes (Table 4). In addition, unexpected duplicate copies of some of the genes, including Fal1 and Mcm7, were discovered. It was therefore concluded that the genomes were complete enough to recover genes of interest, and further to lend confidence to calls of absence were the genes not recovered. It was deemed important to be able to comment on absence since initial attempts to amplify by PCR the MEP α gene from several of the lineages included here consistently failed. Using the four *Cladonia grayi* MEP genes as queries, the MEP genes from all eight genomes were identified (Table 4) as described below.

**Table 4:** Presence/absence of ammonium transporter/ammonia permease genes and 20 conserved test genes

	***Acarospora strigata***	***Arthonia rubrocincta***	***Dibaeis baeomyces***	***Endocarpon pallidulum***	***Graphis scripta***	***Leptogium*austroamericanum**	***Peltula cylindrica***	***Physcia *cf. *stellaris***
***mep1a*** (MEP α)	0	0	X	0	X	0	0	0
***mep1b*** (MEP α)	0	0	X	0	0	0	0	0
***mep2*** (MEP γ, high-affinity)	X	X	X	X	0	X	X	X
***mep3*** (MEP γ, low-affinity)	X	X	X	X	X	X	X	X
***amd1***	X	X	X	X	X	X	X	X
***bub2***	X	X	X	X	X	X	X	X
***cct2***	X	X	X	X	X	X	X	X
***cox15***	X	X	X	X	X	X	X	X
***ctk1***	X	X	X	X	X	X	X	X
***dpb3***	X	X	X	X	X	X	X	X
***eft2***	X	X	X	X	X	X	X	X
***fal1***	X	X	X	X	X	X	X	X
***frs2***	X	X	X	X	X	X	X	X
***gln4***	X	X	X	X	X	X	X	X
***krr1***	X	X	X	X	X	X	X	X
***mcm7***	X	X	X	X	X	X	X	X
***ost1***	X	X	X	X	X	X	X	X
***ret1***	X	X	X	X	X	X	X	X
***rio1***	X	X	X	X	X	X	X	X
***rpa135***	X	X	X	X	X	X	X	X
***rpb1***	X	X	X	X	X	X	X	X
***rpb2***	X	X	X	X	X	X	X	X
***rpo31***	X	X	X	X	X	X	X	X
***sec15***	X	X	X	X	X	X	X	X

### Plant-like MEP α AMTPs are missing from the genomes of lichens growing in high-nitrogen habitats

**Figure 1:**
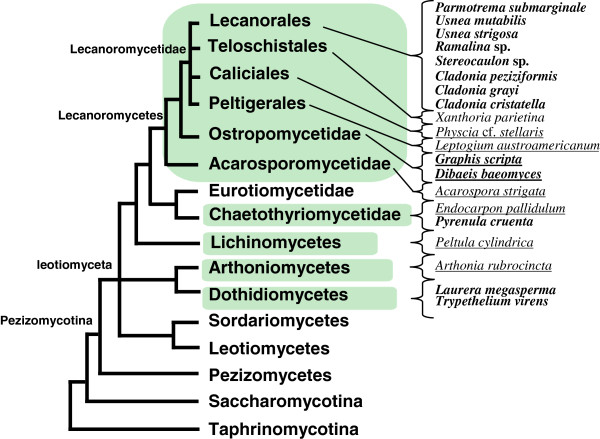
**Simplified phylogenetic tree showing the placement of the eight lichenizing fungi used for genome sequencing.** Lichenizing fungi used for genome sequencing are underlined. Species with plant-like transporters in the MEP α clade are shown in bold. Simplified from [56,69].

Previously, the MEP α genes had been amplified from the Lecanorales but not from any other order sampled within the subclass Lecanoromycetidae (Figure 1). The genomes of lichens in two additional orders within the Lecanoromycetidae, the Caliciales (*sensu*[59]) and the Peltigerales, were sequenced here. One more order, the Teloschistales, is now represented by a genome of the lichenizing fungus *Xanthoria parietina*[68]. These orders are particularly interesting in terms of nitrogen tolerance and acquisition. Lichens in the Teloschistales and Caliciales tend to grow in nitrogen-rich environments, such as rocks or tree limbs on which birds perch. Because nitrogen is not limiting in these environments, it was hypothesized that the previous failure to amplify by degenerate PCR any MEP α genes in the lichens in these orders was evidence that the plant-like AMTPs genes had been lost. As predicted, no plant-like AMTP was found in *Physcia* cf. *stellaris* (Table 4). The same was true for *Xanthoria parietina*. The loss of plant-like AMTPs in the lichens in these orders might suggest why many of these lichens are constrained to living in high-nitrogen environments; alternatively, the expansion of lichens in the Teloschistales and Caliciales into high-nitrogen niches may have removed the selective pressure to retain the MEP α genes.

### Plant-like MEP α AMTPs are missing from the genomes of lichens that are symbiotic with nitrogen-fixing cyanobacteria

Another order of lichens within the Lecanoromycetidae for which no plant-like AMTPs were amplified by degenerate PCR is the Peltigerales. This order is primarily composed of lichens in which the photobiont is a nitrogen-fixing cyanobacterium (*Nostoc*). Also in this order are lichens with both a green alga and a cyanobacterium as photobionts, but these lichens have secondarily regained the green algal photobiont [58]. Data from two unpublished genome projects on various species of *Peltigera* showed that each species of *Peltigera* had high- affinity fungal AMTPs and one low-affinity fungal AMTP, but no plant-like AMTPs (Ólafur S. Andrésson, personal communication and unpublished data; Bernard Goffinet, unpublished data). The fact that one lichen genus that is symbiotic with a nitrogen fixer seemed to have lost the plant-like AMTPs led to the hypothesis that cyanolichens in general have lost the MEP α AMTPs. To test this hypothesis, the lichen *Leptogium* austroamericanum in a separate suborder of the Peltigerales was chosen for genome sequencing. No MEP α AMTPs were found (Table 4). Thus, because lichens in both suborders of the Peltigerales lack the plant-like AMTPs, we suggest that all the lichens in the Peltigerales lack the plant-like AMTPs. Previous studies have reconstructed the ancestral state of the Peltigerales as a bi-membered association with a cyanobacterium [58]. It is possible that, having acquired a nitrogen fixer as a symbiont, the need for the nitrogen presumably provided by the horizontally transferred AMTPs was alleviated, allowing this gene to be lost during the early evolution of the Peltigerales. This suggests that the MEP α AMTPs will not be found in any of the green-algal associated members of the order either, as the symbiosis with a green alga is secondarily acquired. This may also suggest a reason why even most green algal members of the order continue to retain cyanobacteria in cephalodia—to supplement the nitrogen budget. Likewise, it is unusual for a fungus to have just two AMTP genes, particularly a filamentous ascomycete. Perhaps the presence of a nitrogen-fixing symbiont provides sufficient nitrogen so that there is no pressure to duplicate the AMTPs. In addition to the Peltigerales, cyanolichens are also found in the Lichinomycetes, a small class of lichenizing fungi evolutionarily distant from the bulk of lichens found in the Lecanoromycetes (Figure 1). Lichen-forming fungi in the Lichinales are mostly symbiotic with cyanobacteria other than *Nostoc*, such as *Anacystis*[70]. It was hypothesized that, like the lichens in the Peltigerales, lichens in the Lichinomycetes would also have lost the MEP α AMTPs upon acquisition of a cyanobacterium as a photobiont. No MEP α AMTPs were found in this genome. It seems likely that the other members of this class which are also symbiotic with cyanobacteria may also have lost the MEP α AMTP.

### Plant-like AMTPs are found in the Ostropomycetidae but not the Acarosporomycetidae, and are distributed patchily in the Chaetothyriomycetidae

If loss of MEP α AMTPs is strictly associated with the photobiont of a lichen, then it would be expected that lichens with green algal photobionts should retain the MEP α gene(s). However, the failure to identify MEP α genes in other subclasses within the Lecanoromycetes by degenerate PCR argued against this hypothesis. To determine whether MEP α genes were present in other subclasses within the Lecanoromycetes, namely the Ostropomycetidae and the Acarosporomycetidae (Figure 1), the genomes of *Dibaeis baeomyces* (Ostropomycetidae), *Graphis scripta* (Ostropomycetidae) and *Acarospora strigata* (Acarosporomycetidae) were sequenced. MEP α genes were identified in *Graphisscripta* and *Dibaeis baeomyces,* both members of the Ostropomycetidae (Table 4, Figure 1). This finding represents an extension of the known distribution of MEP α genes. Further, *Dibaeis* and *Graphis* are in different orders within this subclass, suggesting that the MEP α gene may be widespread in the subclass (Figure 1). No plant-like AMTPs were found in the genome of *Acarospora strigata*, which is found in the earliest diverged subclass within the Lecanoromycetidae. Previously, one lichen in the subclass Chaetothyriomycetidae (*Pyrenula cruenta*) was shown to have a MEP α gene. Genome sequencing of *Endocarpon pallidulum*, a second lichen in the subclass Chaetothyriomycetidae but in a different order, revealed that no MEP α AMTP was present in the genome. This was surprising as *Trypethelium virens* and *Laurera megasperma* in the Dothidiomycetes, even more distantly related class, were shown to have the MEP α genes [10].

### The MEP α gene replaces the high-affinity MEP γ gene in *Graphis scripta*

Low-affinity fungal AMTPs falling into the MEP γ clade were found in all genomes, as expected given the presence of these genes in the genomes of all Dikarya fungi sequenced to date (Table 4). Likewise, high-affinity fungal AMTPs in the MEP γ clade have been found in all fungi to date [10]. However, high-affinity fungal AMTPs were recovered in only seven of the eight lichen genomes sequenced here. *Graphis scripta* appeared to be lacking a high-affinity MEP γ gene, which is unprecedented among the Dikarya fungi. The similarity of MEP genes is high throughout the transmembrane domains, such that any MEP gene used as a query will find all other MEP genes in the genome. Furthermore, the *Graphis scripta* genome was produced from the cultured fungus, and the genome is very high coverage (173×). Of the eight new genomes presented here, this is the genome that received the highest CEGMA score for complete genes, a score nearly identical to the CEGMA score obtained by the *Neurospora crassa* genome (Table 1). Therefore, it is unlikely that no portion of the high-affinity MEP γ gene would have been sequenced if it were in the genome. Thus, it seems likely that this fungus actually lacks the high-affinity MEP γ gene.

Other lichens like *Arthoniarubrocincta* or *Leptogium* austroamericanum that have only two AMTPs have the fungal high-affinity MEP γ gene and the fungal low-affinity MEP γ gene (Table 4). *Dibaeis baeomyces*, which like *Graphis scripta* is in the Ostropomycetidae, has two copies of a MEP α gene as well as one fungal high-affinity AMTP and one fungal low-affinity AMTP, suggesting that perhaps the ancestor to the Ostropomycetidae or even the Lecanoromycetes had all four AMTPs. It would appear that in *Graphis scripta* the MEP α gene has replaced the high-affinity fungal AMTP from the MEP γ clade. *Graphis scripta* has a very thin crustose thallus, quite unlike the large thalli produced by many lichens in the Lecanorales. It is perhaps no surprise that less nitrogen is needed to support this comparatively reduced thallus. That the MEP α gene is retained in preference to the high-affinity fungal AMTP suggests that it was perhaps more efficient than the high-affinity fungal AMTP.

### The MEP genes are functional AMTPs

**Figure 2:**
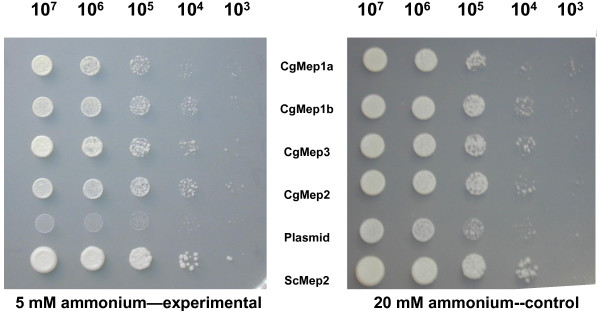
**Heterologous expression of *Cladonia grayi *AMTP genes in a strain of *Saccharomyces cerevisiae *lacking AMTPs.** Left: Ten-fold serial dilutions (10^7^ to 10^3^ cells/ml) of a *S. cerevisiae* (Sc) AMTP triple knockout complemented with AMTPs from the lichenizing fungus *Cladonia grayi* (Cg). Left, limiting ammonium (5 mM); right, control plate with excess ammonium (20 mM). Top to bottom, 1–6, transformants of MLY131 (a/α) carrying a centromeric plasmid (p416-GPD) containing the following genes expressed the constitutive GPD promoter: 1. CgMep1a, 2. CgMep1b, 3. CgMep3, 4. CgMep2, 5. Plasmid only (negative control) 6. *Saccharomyces cerevisiae* Mep2 (positive control).

The fungal high-affinity AMTP (*mep2*), fungal low-affinity AMTP (*mep3*), and two plant-like AMTPs (*mep1a* and *mep1b*) were introduced individually into a strain of *Saccharomyces cerevisiae* in which all three native AMTPs had been deleted. All four *C. grayi* genes complemented the mutant, although growth was not robust. Therefore, each of the genes was deemed to be a functional AMTP (Figure 2). Of the four genes, the low-affinity AMTP (*mep3*) appeared generated the most robust growth under the conditions tested, albeit marginally so. This observation is consistent with the predicted function of this gene as a low-affinity (high-capacity) AMTP. Importantly, the *mep1a* and *mep1b* are the first genes from the fungal subclade of the MEP α clade for which a function has been demonstrated.

## Discussion

Nitrogen is an important currency in the cell, arguably second in importance only to carbon. A recent work on fungal genomes has validated 323 horizontal gene transfer events into fungi [71] of which the two top categories are genes involved in the acquisition and metabolism of carbon and nitrogen. If all the categories involving nitrogen are grouped, horizontal gene transfers of genes involved in nitrogen acquisition and metabolism are in fact the most populous category. Evidently fungi have been capturing new technology for nitrogen acquisition since the very beginning.

AMTPs in particular show an intriguing pattern of expansion in the fungi. The microsporidia, considered to be the earliest diverging fungi [69], lack AMTPs. Interestingly, the microsporidia are intracellular pathogens. Intracellular pathogens tend to experience genome contraction as they outsource more and more of their life functions to their hosts. If other early fungi also lack AMTP genes, it would lend support to the hypothesis that fungi first lost the eukaryotic AMTPs and then obtained by horizontal gene transfer a prokaryotic AMTP. In fact, the seemingly high levels of horizontal gene transfer seen in the fungi could be explained similarly, as fungi slowly rebuilding their genomic toolkits after a period of gene loss as intracellular pathogens during their early evolution.

After a period of time with no AMTPs, fungi acquired by horizontal gene transfer a bacterial AMTP. Sampling of the Neocallimastigomycota and the Monoblephardiomycetes (chytrids *sensu lato*) is poor, so it isn’t yet possible to pinpoint the entry of AMTPs into the fungi, but AMTPs had entered the fungi at least by the time of the divergence of the chytrid *Batrachochytrium*. Then, as the Dikarya diverged there was a duplication event followed by a subfunctionalization into high-affinity (low-capacity) and low-affinity (high-capacity) AMTPs. In mycorrhizal basidiomycetes (but not rusts or smuts), a gene family expansion followed. In ascomycetes, there may also have been a slow gene family expansion perhaps starting as early as the divergence of the Taphrinomycotina. Layered on top of this slow gene family expansion was a second horizontal transfer of the MEP α AMTP from hyperacidophilic chemoautolithotrophic prokaryotes into the leotiomyceta [10].

MEP α has been subsequently lost in almost all non-lichenized lineages [10] and, as shown here, in certain lichenized lineages. What the lichenized lineages that have lost the plant-like MEP α have in common with each other is not entirely clear. At the outset of this work, it was hypothesized that nitrogen availability was the major factor governing the retention or loss of the MEP α genes. It was expected that lichens with a rich enough internal or external source of nitrogen would have lost the MEP α genes, while other lichens would retain it. While this appears to hold true within the Lecanoromycetes with the current sampling, this hypothesis cannot explain the apparent loss of the genes in *Endocarpon pallidulum* (Chaetothyriomycetidae) or *Arthoniarubrocincta* (Arthoniomycetes). Since retention of the AMTPs is not solely dependent on low nitrogen availability, evidently some other factors govern the loss of AMTPs.

Because the MEP α genes are missing from some lichens, these genes may not be intimately involved in the lichen symbiosis, assuming that there is some ancestral core “symbiosis program” shared by ascomycete lichens, nor are the MEP α genes absolutely mandatory to supplying nitrogen to the lichenizing fungus. It is possible however, that the MEP α genes are involved specifically in balancing the nitrogen budget between the fungal and green algal symbionts. If lichenization is considered a controlled parasitism of the photobiont by lichenizing fungi, it follows that the fungus would also control the nitrogen budget of the alga, perhaps by outcompeting the photobiont for ammonium but then exporting, for example, amino acids or some other form of nitrogen on which the alga is dependent. If so, this would predict that large lichens that live on nitrogen-poor substrates, such as *Umbilicaria* (Lecanoromycetes, uncertain placement) or *Dermatocarpon* (Chaetothyriomycetidae) should have retained the MEP α AMTPs, while explaining why smaller lichens on nitrogen-poor substrate like the aforementioned *Arthonia* and *Endocarpon* have lost them. Of course, the alga might also scavenge its own ammonium, and may in fact be fed by the fungus releasing ammonium through ammonium exporters (e.g. those encoded by the *ATO* genes in yeast).

Perhaps the MEP α genes have been retained for a reason other than nitrogen acquisition. Aside from transporting ammonium/ammonia, AMTP proteins (specifically, only high-affinity fungal AMTPs) have also been shown to be involved in pseudohyphal filamentation in *Saccharomyces cerevisiae* and in *Cryptococcus neoformans*, where they serve as sensors of nitrogen starvation [72,73]. High-affinity AMTPs from the basidiomycetes *Ustilago maydis* and *Hebeloma cylindrosporum* can complement this function in strains of *Saccharomyces cerevisiae* with the high-affinity AMTP *Mep2* deleted [31,74]. Presumably, the high-affinity fungal AMTP from *C. grayi* should also complement this function, while the low-affinity fungal AMTP should not, in keeping with the functional data from other fungi. It is not yet known whether the MEP α AMTPs are involved in sensing nitrogen, nor is it known whether they can stimulate pseudohyphal filamentation, since they are high-affinity AMTPs, or in a perhaps more likely scenario, will fail to do so due to amino acid sequence differences in regions shown to be critical for pseudohyphal filamentation [72].

In order to determine the function of the new clade of AMTPs, transcriptional profiling of the fungus *C. grayi* grown on nitrogen-rich or nitrogen-poor media would be helpful. Identifying genes co-regulated with the MEP α AMTPs could reveal whether MEP α AMTPs are involved in mating, morphological change, growth, nutrient scavenging, or other pathways. Understanding this could shed light on why these genes have been retained in some lichenizing fungi but not others, and why these genes were horizontally transferred in the first place.

## Conclusions

Over evolutionary time, lichenizing fungi have retained the MEP α AMTPs except if an environmental or symbiotic source of nitrogen is available. Additional conditions precipitating the loss of the MEP α AMTPs exist and remain unelucidated.

## Methods

### Media and culture conditions

For yeast strains, standard yeast media were used including: Synthetic Limiting Ammonium and Dextrose (SLAD) composed of 0.17% yeast nitrogen base (without amino acids and ammonium sulfate), 2% glucose and 2% agar [72,75] with modifications including 2 mM, 5 mM, 7 mM, 10 mM and 20 mM ammonium sulfate; and Synthetic Complete medium (SC) lacking uracil, which contains 6.7 g/l yeast nitrogen base without amino acids, supplemented with all amino acids except uracil, 2% glucose and 2% agar. Yeast strains were grown at 30°C or at room temperature. *Cladonia grayi* was maintained at room temperature in liquid shaking cultures of MEYE medium consisting of 20 g/l malt extract and 2 g/l yeast extract [1].

### Construction of plasmids

RNA was extracted from *Cladonia grayi* cultures growing in liquid medium. Tissue from liquid cultures was harvested, rinsed in distilled water, lyophilized, then ground to a fine powder under liquid nitrogen in a pre-chilled mortar and pestle. The ground tissue was resuspended in TRIzol Reagent (Invitrogen, Carlsbad, CA) and RNA extraction proceeded according to the manufacturer’s instructions.

The first strand of the cDNA was generated using the following reverse transcription reaction mix: 1.0 μl 10× PCR buffer, 2.0 μl 25 mM MgCl2 (both supplied with AmpliTaq DNA Polymerase, Applied Biosystems), 2.0 μl dNTPs (10 mM each), 0.5 μl RNAse inhibitor (20 μg/μl, Applied Biosystems) 0.5 μl MulV reverse transcriptase (Applied Biosystems), 1.75 μl of distilled water, and 1.25 μl reverse primer. The thermocycling conditions for this first strand synthesis reaction were: 42°C for 35 minutes, then 99°C for 5 minutes. Primers are listed in Additional files 1 and 2. PCR to regenerate the second strand of the cDNA was performed using the entire volume of first strand reaction plus 31.25 μl distilled water, 4 μl 20x PCR buffer, 3 μl 25 mM MgCl2, 1.25 μl of a forward primer, 0.25 μl of AmpliTaq polymerase (Applied Biosystems) and 0.25 μl of an antibody to Taq polymerase (Clontech). The thermocycling program consisted of 24 cycles of 94°C for 30 seconds, 55°C for 30 seconds with a 0.4 degree decrease in temperature for each cycle, and 72° for 1 minute, followed by 12 cycles of 94°C for 30 seconds, 45°C for 30 seconds, and 72°C for 2 minutes with a 3 second increase per cycle, followed by a final extension at 72°C for 10 minutes. cDNA of each AMTP from *Cladonia grayi* was cloned into the TOPO TA vector as described above. Plasmid DNA was restricted to release the full-length AMTP cDNA which was then subcloned into the pRS306-GAL1-TADH vector (unpublished, a gift from Mark Chee) carrying a uracil selectable marker, an inducible Gal promoter and a Cyc terminator or the p416-GPD vector [76] carrying a uracil selectable marker and a constitutive GPD promoter and Cyc terminator, for transformation into *Saccharomyces cerevisiae*. The DNA sequence of the constructed plasmids was confirmed by PCR and sequencing as described below.

### Construction of yeast strains

Yeast strains are listed in Additional file 3. Yeast transformations were performed using the TRAFO lithium acetate method [77] or by the gapped plasmid method [78]. Following the TRAFO method, a yeast culture in log phase was centrifuged for 5 minutes at 6000 RPM, and the pellet washed with distilled water, and resuspended in an equal volume of distilled water, pelleted again, then resuspended in 1 ml of 100 mM lithium acetate. This solution was pelleted at maximum speed (>14,000 RPM) in a microcentrifuge and the pellet resuspended in approximately 400 μl of 100 mM lithium acetate. A transformation mix of 50 μl yeast cells, 240 μl PEG, 36 μl 1 M lithium acetate, 50 μl salmon sperm DNA and 34 μl of diluted plasmid (approximately 100 ng plasmid) was incubated at 30°C for 30 minutes and heat shocked at 42°C for 30 minutes. The mix was then centrifuged and the pellet resuspended in 300 μl water. 100 μl of the cell suspension was plated onto selective medium and transformants confirmed by colony PCR as described below. For gapped plasmid construction, cDNA of AMTPs from *C. grayi* which had been cloned into the TopoTA vector (Invitrogen, Carlsbad, CA) was amplified with primers composed of 40 base pairs of homology on either side of the multiple cloning site of the centromeric plasmid p416GPD [76] carry the uracil selectable marker and joined to the first or last 20 base pairs of the gene [78]. The PCR product was cleaned in a Microcon column (Millipore, Bilerica, MA) and approximately 1 μg of amplicon was transformed into yeast along with 0.1 μg of restricted plasmid DNA. Yeast recombine the two fragments into a functional plasmid. Transformants were screened by PCR and the inserts sequenced as described below.

### DNA amplification and Sanger Sequencing

For PCR performed directly on colonies of yeast or *E. coli*, the PCR reaction mix consisted of 17.3 μl distilled water, 2.5 μl of 10x PCR buffer, 2.5 μl dNTPs, 1.25 μl each of a forward primer and a reverse primer, and 0.3 μl Taq polymerase (Denville). Colonies were touched lightly with the tip of a pipette which was then dipped briefly into the PCR tube and removed. PCR was performed on a MJ Research PTC200 thermocycler or an Applied Biosystems (Foster City, CA) Veriti thermocycler using a cycling program consisting of 10 minutes at 94°C to lyse the cells and release the DNA, followed by 25 cycles of 30 seconds at 94°C, 30 seconds at 55°C and 2 minutes at 72°C, followed a final elongation step at 72°C for 7 minutes. PCR products were visualized on a TAE 1% agarose gel stained with SYBR Safe (Invitrogen, Carlsbad, CA). If necessary, faint products or products with multiple bands were cloned with the TOPO TA cloning kit (Invitrogen, Carlsbad, CA) following the manufacturer’s instructions. For each cloning reaction, at least 8 clones were screened by colony PCR using T7 and M13R primers and a PCR program consisting of a 10-minute initial denaturation step, followed by 25 cycles of 30 seconds at 94°C, 30 seconds at 52°C and 60 seconds at 72°C followed by a final elongation step of 7 minutes. PCR products were cleaned with a Montage PCR filter column (Millipore, Bilerica, MA) or with an Exo-SAP clean-up using 1 μl SAP dilution buffer, 0.5 μl Exonuclease 1, 0.5 μl Shrimp Alkaline Phosphatase, added to 10 μl PCR reaction and incubating on one of the aforementioned thermocyclers for 30 minutes at 37°C, then 15 minutes at 80°C. Cleaned PCR products were sequenced in 10 μl reactions using: 1 μl primer, 3 μl purified PCR product, 0.5 μl Big Dye (Big Dye Terminator Cycle sequencing kit, ABI PRISM version 3.1; PE Applied Biosystems, Foster City, CA), 1.5 μl Big Dye buffer, and 4 μl double-distilled water. Automated reaction clean-up and visualization was performed at the Duke IGSP Genome Sequencing & Analysis Core Facility using Big Dye chemistry with an ABI 3730xl automated sequencer (PE Applied Biosystems, Foster City, CA). Sequencher version 4.8 (Gene Codes Corporation, Ann Arbor, MI) was used to edit sequences and assemble contigs.

### Genomic DNA isolation

DNA from cultures of the lichenizing fungi *Acarospora strigata* (Acarosporomycetidae, Lecanoromycetes), *Arthoniarubrocincta* (Arthoniomycetidae), *Endocarpon pallidulum* (Eurotiomycetes) *Graphis scripta* (Ostropomycetidae, Lecanoromycetes) and from lichen thalli of *Dibaeis baeomyces* (Ostropomycetidae, Lecanoromycetes), *Leptogium* austroamericanum, *Physcia* cf. *stellaris* (both Lecanoromycetidae, Lecanoromycetes), and *Peltula cylindrica* (Lichinomycetes) was prepared following the DTAB/CTAB method outlined in [79]. Briefly, tissue was ground to a fine powder under liquid nitrogen using a pre-chilled mortar and pestle. Twenty volumes of a DTAB solution (with 1% DNAse-free RNAse) was added to the powder and incubated for ~3 minutes at 65°C. Polysaccharides and other contaminants were precipitated by adding 1/3 volume 5M NaCl and centrifuging at 6000 RPM for 5 minutes. The supernatant was removed and extracted once with one volume of phenol, after which the supernatant was removed and then extracted with one volume of chloroform. The supernatant was removed, and DNA precipitated with one volume of isopropanol and left to incubate at room temperature for 5 minutes, then centrifuged at 6000 RPM for 5 minutes to pellet the DNA. The pellet was resuspended in DTAB + RNAse, with heating and gentle agitation. Remaining polysaccharides were precipitated by adding 1/3 volume of 5M NaCl and centrifuging at 6000 RPM for 5 minutes. The supernatant was removed and undissolved material was pelleted by centrifugation at 6000 RPM. DNA was precipitated by adding 2 volumes of 100% ethanol, incubating at room temperature for five minutes, and centrifuging at maximum speed (14,000 RPM) for five minutes. The pellet was washed once with 70% ethanol, and allowed to dry. When dry, the pellet was resuspended in 100 μl TE and quantified by Qubit. DNA was then further purified using the PowerClean kit (Mo Bio) following the manufacturer’s instructions. DNA was again quantified after purification by Qubit.

### Illumina sequencing and genome assembly

One to six μg of purified DNA were submitted to the sequencing facility at Duke University for 75-base pair paired-end barcoded Illumina sequencing using the HiSeq technology. Four lichen cultures were multiplexed onto a lane, and DNA from two lichen thalli (yielding both fungal and algal DNA) were multiplexed onto a lane. A total of three lanes were used to sequence the eight fungal genomes (~80 million to 600 million reads). Preliminary draft assemblies were generated with Velvet [80] (Table 1). Data is housed in the Sequence Read Archive (SRA) accessible through the National Center for Biotechnology Information (NCBI) website.

### Phylogenetic analysis

AMTP sequences generated for this study (Additional file 4) were included in a modified alignment from [10] and phylogenetic analysis was performed as in [10]. Genes in the MEP clade of AMTPs were retained in the analysis (Additional file 5), while all others were discarded. All excluded regions were reexamined, and additional sites that became alignable were included in the analysis, including 90 base pairs of transmembrane region one, which in the previous analysis had been entirely excluded. The first well-supported clade outside of the MEP group in the MEP grade was used as an outgroup. In one analysis, the three AMTPs from the green alga *Asterochloris* sp., which fall into the AMT clade of AMTPs, were also included and used as an outgroup. Manual alignments were performed using MacClade 4.08 [81]. Ambiguously aligned regions and introns were delimited manually and excluded from phylogenetic analyses. Models of molecular evolution which had been selected using the Akaike Information Criterion (AIC) implemented in jModeltest [82] or MrModeltest 2.3 [83] were implemented on this dataset. Phylogenetic relationships and confidence values were inferred using a maximum likelihood approach at the nucleotide level. Maximum likelihood analysis at the nucleotide level used GTR GAMMAI (with a gamma parameter and a proportion of invariable sites, = GTR + Γ + I). The program RAxML-VI-HPC [84] was used for the maximum likelihood search for the most likely tree. The same program using the same settings was used for the bootstrap (BS) analysis with 1000 BS replicates run in batches of 100 replicates and pooled. Bootstrap values were calculated and visualized using the majority rule consensus tree command in PAUP 4.0d701 [85] (Additional file 6).

## Abbreviations

AMTPs: Ammonium transporters/Ammonia permeases; MEP: Methylammonium permease.

## Competing interests

The authors declare that they have no competing interests.

## Authors’ contributions

TM carried out the molecular genetic studies, the phylogenetic analyses, sequenced the genomes, performed bioinformatics analyses, and drafted the manuscript. OM performed genome assemblies and downstream bioinformatics analyses. FD assisted in strain construction and bioinformatics. FL critically reviewed the manuscript and was the main advisor of TM for her Ph.D. from which this article is derived. All authors read and approved the final manuscript.

## Supplementary Material

Additional file 1 PCR and sequencing primers (5’ to 3’). Click here for file

Additional file 2 Gapped plasmid construction plasmids. Click here for file

Additional file 3 **Strains of *Saccharomyces cerevisiae *carrying AMTP genes from *Cladonia grayi ***[86].
Click here for file

Additional file 4 Accession numbers for AMTP genes from lichen genomes. Click here for file

Additional file 5 Accession numbers and genome coordinates of ammonium transporter/ammonia permease genes included in the phylogenetic tree. Click here for file

Additional file 6 **Phylogenetic placement of ammonium transporters/ammonia permeases from eight lichen genomes.** Maximum likelihood analysis of 300 ammonium transporter/ammonia permease genes details the phylogenetic placement of ammonium transporter/ammonia permease genes in the well-supported predominantly prokaryotic clade (MEP) in which eukaryotic lineages demonstrate horizontal gene transfer. Click here for file
