# Supplementary material for: High-throughput genome sequencing of lichenizing fungi to assess gene loss in the ammonium transporter/ammonia permease gene family
Source: BMC Genomics. 2013 Apr 4;14:225. doi: 10.1186/1471-2164-14-225 (PMC3663718; doi:10.1186/1471-2164-14-225)
Supplement: Additional file 1 — PCR and sequencing primers (5’ to 3’). [file 1471-2164-14-225-S1.pdf]

Additional file 1 - PCR and Sequencing primers (5’ to 3’)

CgrayiA_F	CAATACTCACCATGGCTACCAAC
CgrayiA_R	GAAGCATTGGGTATCTCAACAAA
CgrayiAmtpC_for_int	AGGTAAATTGCCCGAGTCCT
CgrayiAMTpC_for_int2	CGATTCTTGACTGATTGCACA
CgrayiAmtpC_rev_int	GTTTATGAGGAAGGCGAGGA
CgrayiAmtpC_rev_int2	ATTGTGAGGACGGAAGTTGG
CgrayiAmtpD_for_int	AGACCGAGGCTAACGATGTG
CgrayiAmtpD_for_int2	CTCATGCTGGGAAAGAGGAG
CgrayiAmtpD_rev_int	GATGCCGAGAAGGAATTTGA
CgrayiAmtpD_rev_int2	TCCATGTCCAGCAAGCAATA
CgrayiB_F	CAATACTCACCATGGCTACCAAC
CgrayiB_R	GAAGCATTGGGTATCTCAACAAA
CgrayiC_F	TACTAAGCCAACCGGTACCTCTG
CgrayiC_R	CCCTCCCCCTACCCATGC
CgrayiD_F	CACAACAAGATGGCTTCAGGAC
CgrayiD_R	CGATGACAGTGTCGTAGCATTTC
MepA_F_nest2	CTTCTTGGTCTTGGCTCTGG
MepA_F_nest3	CTGTGCTGCTTTCGGTATCA
MepARev_nest1	TGATACCGAAAGCAGCACAG
MepArev_nest2	TGCTGCTACAAAACCAGCAG
