# Supplementary material for: High-throughput genome sequencing of lichenizing fungi to assess gene loss in the ammonium transporter/ammonia permease gene family
Source: BMC Genomics. 2013 Apr 4;14:225. doi: 10.1186/1471-2164-14-225 (PMC3663718; doi:10.1186/1471-2164-14-225)
Supplement: Additional file 2 — Gapped plasmid construction plasmids. [file 1471-2164-14-225-S2.pdf]

Additional file 2 - **Gapped plasmid construction plasmids**

CgrayiA_p416F	AGTTTTAAAACACCAGAACTTAGTTTCGACGGATTC TAGAATGTCGTCCACCATCGCTGC
CgrayiA_p416R	CGATAAGCTTGATATCGAATTCCTGCAGCCCGGGGG ATCCTTACTCTGTTTTCTTGGAGT
CgrayiA_PgalF	TCTATACTTTAACGTCAAGGAGAAAAAACCCCGGA TCCACATGTCGTCCACCATCGCTGC
CgrayiA_PgalR	TCATAAATCATAAGAAATTCGCTTATTTAGAAGTTC TAGATTACTCTGTTTTCTTGGAGT
CgrayiC_p416F	AGTTTTAAAACACCAGAACTTAGTTTCGACGGATTC TAGAATGACGAGCTATCCCGCTCC
CgrayiC_p416R	CGATAAGCTTGATATCGAATTCCTGCAGCCCGGGGG ATCCCTACCCATGCGCCTGCCCCT
CgrayiC_PgalF	TCTATACTTTAACGTCAAGGAGAAAAAACCCCGGA TCCACATGACGAGCTATCCCGCTCC
CgrayiC_PgalR	TCATAAATCATAAGAAATTCGCTTATTTAGAAGTTC TAGACTACCCATGCGCCTGCCCCT
CgrayiD_PgalF	TCTATACTTTAACGTCAAGGAGAAAAAACCCCGGA TCCACATGGCTTCAGGACCGGTGCT
CgrayiD_PgalR	TCATAAATCATAAGAAATTCGCTTATTTAGAAGTTC TAGACTACCTCAAAGGCCCCCTCA
p416_D_F	AGTTTTAAAACACCAGAACTTAGTTTCGACGGATTC TAGAATGGCTTCAGGACCGGTGCT
p416_D_R	TGAGGGGGCCTTTGAGGTAGCCCCCGGGCTGCAGG AATTCGATATCAAGCTTATCG
p416_ScMep2_F	AGTTTTAAAACACCAGAACTTAGTTTCGACGGATTC TAGAATGTCTTACAATTTTACAGG
P416_ScMEp2_R	CGATAAGCTTGATATCGAATTCCTGCAGCCCGGGGG ATCCTTATACTATATGGTCAGTGT
P416B_F	AGTTTTAAAACACCAGAACTTAGTTTCGACGGATTC TAGAATGGCTACCAACCCACCATG
p416B_R	CGATAAGCTTGATATCGAATTCCTGCAGCCCGGGGG ATCCTTAATCATGCTTTATCTCCC

p416F	CAGAACTTAGTTTCGACGGATTC
P416R	CGATAAGCTTGATATCGAATTCCT
