# Supplementary material for: High-throughput genome sequencing of lichenizing fungi to assess gene loss in the ammonium transporter/ammonia permease gene family
Source: BMC Genomics. 2013 Apr 4;14:225. doi: 10.1186/1471-2164-14-225 (PMC3663718; doi:10.1186/1471-2164-14-225)
Supplement: Additional file 3 — Strains of Saccharomyces cerevisiae carrying AMTP genes from Cladonia grayi [86]. [file 1471-2164-14-225-S3.docx]

**Additional file 3 - Strains of *Saccharomyces cerevisiae* carrying AMTP genes from *Cladonia grayi*.** All strains are congenic to Σ1278b* except S288C. All lichen genes are expressed from the p416-GPD plasmid unless otherwise noted.

| **Strains**  **(mating type)** | **Genotype** | **References**  **and comments** |
| --- | --- | --- |
| MLY131 (a) | ∆*mep1::LEU2,* ∆*mep2*::*LEU2,* ∆*mep3::G418*, *ura3-52*, *MAT* a | Lorenz and Heitman, 1998 |
| MLY131 (α) | ∆*mep1::LEU2*, ∆*mep2*::*LEU2*, ∆*mep3::G418*, *ura3-52*, *MAT α* | Lorenz and Heitman, 1998 |
| FD131 (a/α) | ∆*mep1::LEU2/*∆*mep1::LEU2*, ∆*mep2*::*LEU2/*∆*mep2*::*LEU2*, ∆*mep3::G418/*∆*mep3::G418*,  *ura3-52/ ura3-52*, *MAT*a | MLY131(a) x MLY131(α) |
| TRM Mep1a (a) | ∆*mep1::LEU2/*∆*mep1::LEU2*, ∆*mep2*::*LEU2/*∆*mep2*::*LEU2*, ∆*mep3::G418/*∆*mep3::G418*,  *ura3-52/ ura3-52*, *MAT*a *, ura4-CgMep1a* | Transformant of MLY131(a) |
| TRM Mep1a (a/α) | ∆*mep1::LEU2/*∆*mep1::LEU2*, ∆*mep2*::*LEU2/*∆*mep2*::*LEU2*, ∆*mep3::G418/*∆*mep3::G418*,  *ura3-52/ ura3-52*, *MAT*a*/α, URA4-CgMep1a-CYC* | Transformant of FD131(a/α) |
| TRM Mep1b (a) | ∆*mep1::LEU2/*∆*mep1::LEU2*, ∆*mep2*::*LEU2/*∆*mep2*::*LEU2*, ∆*mep3::G418/*∆*mep3::G418*,  *ura3-52/ ura3-52*, *MAT*a *MATa, ura4::CgMep1b* | Transformant of MLY131(a)  (Expressed from pRS306-GAL1-TADH) |
| TRM Mep1b (a/α) | ∆*mep1::LEU2/*∆*mep1::LEU2*, ∆*mep2*::*LEU2/*∆*mep2*::*LEU2*, ∆*mep3::G418/*∆*mep3::G418*,  *ura3-52/ ura3-52*, *MAT*a *MATa/α, ura4::CgMep1b* | TRMB x MLY 131(α) |
| TRM MepC (a) | ∆*mep1::LEU2/*∆*mep1::LEU2*, ∆*mep2*::*LEU2/*∆*mep2*::*LEU2*, ∆*mep3::G418/*∆*mep3::G418*,  *ura3-52/ ura3-52*, *MAT*a*, ura4::CgMepC* | Transformant of MLY131 |
| TRM MepC (a/α) | ∆*mep1::LEU2/*∆*mep1::LEU2*, ∆*mep2*::*LEU2/*∆*mep2*::*LEU2*, ∆*mep3::G418/*∆*mep3::G418*,  *ura3-52/ ura3-52*, *MATa/α, ura4::CgMepC* | Transformant of FD131(a/α) |
| **Additional file 4 (continued) - Strains of *Saccharomyces cerevisiae* carrying ATMP genes from *Cladonia grayi*.** | | |
|  |  |  |
| TRM MepD (a) | ∆*mep1::LEU2/*∆*mep1::LEU2*, ∆*mep2*::*LEU2/*∆*mep2*::*LEU2*, ∆*mep3::G418/*∆*mep3::G418*,  *ura3-52/ ura3-52*, *MAT*a *ura4::CgMepD* | Transformant of MLY131(a) |
| TRM MepD (a/α) | ∆*mep1::LEU2/*∆*mep1::LEU2*, ∆*mep2*::*LEU2/*∆*mep2*::*LEU2*, ∆*mep3::G418/*∆*mep3::G418*,  *ura3-52/ ura3-52*, *MAT*a *MATa/α, ura4::CgMepD* | Transformant of FD131(a/α) |
| TRM ScMep2 (a) | ∆*mep1::LEU2/*∆*mep1::LEU2*, ∆*mep2*::*LEU2/*∆*mep2*::*LEU2*, ∆*mep3::G418/*∆*mep3::G418*,  *ura3-52/ ura3-52*, *MAT*a *MATa, ura4::ScMep2* | Transformant of MLY131(a) |
| TRM ScMep2 (a/α) | ∆*mep1::LEU2/*∆*mep1::LEU2*, ∆*mep2*::*LEU2/*∆*mep2*::*LEU2*, ∆*mep3::G418/*∆*mep3::G418*,  *ura3-52/ ura3-52*, *MAT*a *MATa/α, ura4::ScMep2* | Transformant of FD131(a/*α)* |
| S288C | Wild type |  |

*Σ1278b is described in Grenson M, Mousset M, Wiame JM, Bechet J: **Multiplicity of the amino acid permeases in *Saccharomyces cerevisiae*. I. Evidence for a specific arginine-transporting system.** *Biochim Biophys Acta* 1966, **127**(2):325–338.
