# Supplementary material for: High-throughput genome sequencing of lichenizing fungi to assess gene loss in the ammonium transporter/ammonia permease gene family
Source: BMC Genomics. 2013 Apr 4;14:225. doi: 10.1186/1471-2164-14-225 (PMC3663718; doi:10.1186/1471-2164-14-225)
Supplement: Additional file 4 — Accession numbers for AMTP genes from lichen genomes. [file 1471-2164-14-225-S4.docx]

**Additional file 4** **- Accession numbers for AMTP genes from lichen genomes**

| **Gene** | **Accession number** |
| --- | --- |
| *Acarospora strigata* A | KC810860 |
| *Acarospora strigata* B | KC810865 |
| *Arthonia* cf. *rubrocincta* A | KC810856 |
| *Arthonia* cf. *rubrocincta* B | KC810866 |
| *Dibaeis baeomyces* A | KC810874 |
| *Dibaeis baeomyces* B | KC810875 |
| *Dibaeis baeomyces* C | KC810861 |
| *Dibaeis baeomyces* D | KC810871 |
| *Endocarpon pallidulum* A | KC810855 |
| *Endocarpon pallidulum* B | KC810862 |
| *Endocarpon pallidulum* C | KC810870 |
| *Endocarpon pallidulum* D | KC810864 |
| *Graphis scripta* A | KC810876 |
| *Graphis scripta* B | KC810854 |
| *Leptogium* sp. A | KC810859 |
| *Leptogium* sp. B | KC810868 |
| *Peltigera membranacea* | KC810863 |
| *Peltigera* *malacea* | KC810873 |
| *Peltula cylindrica* A | KC810858 |
| *Peltula cylindrica* B | KC810872 |
| *Physcia* cf *stellaris* A | KC810857 |
| *Physcia* cf *stellaris* B | KC810867 |
| *Physcia* cf *stellaris* C | KC810869 |
| *Nostoc* sp. from *Leptogium* | KC810877 |
| Cyanobacterium from *Peltula* | KC810878 |
|  |  |
|  |  |
