# Supplementary material for: High-throughput genome sequencing of lichenizing fungi to assess gene loss in the ammonium transporter/ammonia permease gene family
Source: BMC Genomics. 2013 Apr 4;14:225. doi: 10.1186/1471-2164-14-225 (PMC3663718; doi:10.1186/1471-2164-14-225)
Supplement: Additional file 5 — Accession numbers and genome coordinates of ammonium transporter/ammonia permease genes included in the phylogenetic tree. [file 1471-2164-14-225-S5.pdf]

Additional file 5: Accession numbers and genome coordinates of ammonium transporter/ammonia permease genes included in the phylogenetic tree

Organism, gene abbreviation	GI number or GenBank accession number for DNA	Genome Region	GI number for mRNA	Clade
**EUKARYOTES**				
**Green plants (Viridiplantae; green algae and land plants)**				
Arabidopsis thaliana, AtAMT2	GI:240254678	(16039475..16042383)	GI:42569729	MEP α
Cryptomeria japonica	N/A	N/A	GI:114841288	MEP α
Lotus japonicus, LiAMT2	N/A	N/A	GI:15799271	MEPα
*Medicago truncatula, MtAMT*2	N/A	N/A	GI:22900885	MEP α
Micromonas sp. RCC299, 2	GI:255081797	579223..580970	GI:255079969	MEP β
Oryza sativa, OsAMT2;1	GI:28875520	N/A	GI:19909967	MEP α
Oryza sativa, OsAMT2;2	GI:115442598	(35590381..35592277)	GI:28875522	MEP α
Oryza sativa, OsAMT2;3	GI:20160632	109596..111823	GI:20160632	MEP α
Oryza sativa, OsAMT3;1	GI:115442598	37720693..37731081	N/A	MEP α
Oryza sativa, OsAMT3;2	GI:28269486	77711..81059	GI:32985620	MEP α
Oryza sativa, OsAMT3;3	GI:46390229	104575..107816	N/A	MEP α
Ostreococcus lucimarinus CCE9901, 3	GI:145327766	(334523..336339)	GI:145348948	MEP β
Ostreococcus lucimarinus CCE9901, 4	GI:145335532	34720..37072	GI:145356297	MEP β
Physcomitrella patens subsp. patens, 2.1	GI:168004612	1799359..1802286	GI:168004232	MEP α
Physcomitrella patens subsp. patens, 2.2	GI:168058452	(46510..48246)	GI:168058414	MEP α
Physcomitrella patens subsp. patens, 2.3	GI:168035123	(956773..959548)	GI:168052163	MEP α
Physcomitrella patens subsp. patens, 2.4	GI:168052203	(228592..232717)	GI:168035109	MEP α
Physcomitrella patens subsp. patens, 2.5	GI:167999745	(1405866..1407695)	GI:167999617	MEP α
Physcomitrella patens subsp. patens, 2.6	GI:168020770	823947..825718	GI:168020544	MEP α

Additional file 5 (continued): Accession numbers and genome coordinates of ammonium transporter/ammonia permease genes included in the phylogenetic tree

Organism, gene abbreviation	GI number or GenBank accession number for DNA	Genome Region	GI number for mRNA	Clade
Physcomitrella patens subsp. patens, 2.7	GI:168003095	(2804682..2807626)	GI:168003073	MEP α
Picea sitchensis	N/A	N/A	GI:148909475	MEP α
Populus trichocarpa, PtrAMT2;1	GI:116256301	(7169018..7171902)	GI:224091027	MEP α
Populus trichocarpa, PtrAMT2;2	GI:116256311	(11521306..11523746)	GI:224140458	MEP α
Populus trichocarpa, PtrAMT3;1	GI:116256298	(26343416..26346328)	GI:224060484	MEP α
Populus trichocarpa, PtrAMT4;1	GI:116256317	(3007991..3009863)	GI:224066328	MEP α
Populus trichocarpa, PtrAMT4;2	GI:116256313	5044547..5046163	GI:224141842	MEP α
Populus trichocarpa, PtrAMT4;3	GI:116256320	14994950..14996903	GI:224082701	MEP α
Populus trichocarpa, PtrAMT4;4	GI:116256308	2765333..2767047	GI:224123403	MEP α
Populus trichocarpa, PtrAMT4;5	GI:116265052	(87989..89560)	GI:224133945	MEP α
Selaginella moellendorffii, 2.1[J]	scaffold 37	(1469550-1470973)	N/A	MEP α
Selaginella moellendorffii, 2.2[J]	scaffold 62	(1059095-1060581)	N/A	MEP α
Selaginella moellendorffii, 2.3[J]	scaffold_28	1038805-1040650	N/A	MEP α
Vitis vinifera, 2.1	gi157357314	580580..583260	N/A	MEP α
Vitis vinifera, 2.2	gi157351961	1965726..1969581	N/A	MEP α
Vitis vinifera, 2.3	GI:157339171	4545828..4548058	N/A	MEP α
Vitis vinifera, 2.4	gi157347782	2402288..2404788	N/A	MEP α
Vitis vinifera, 2.5	gi157351961	1965726..1969581	N/A	MEP α
Vitis vinifera, 2.6	gi157329134	88960..90484	N/A	MEP α
Vitis vinifera, 2.7	gi157344876	468779..471094	N/A	MEP α
Vitis vinifera, 2.8	gi157344876	487711..495387	N/A	MEP α
**Fungi**				
Aspergillus fumigatus, meaA	GI:71025129	1654974..1656856	GI:70989734	MEP γ
Aspergillus fumigatus, mep2	GI:71025128	(2853321..2854904)	GI:70995403	MEP γ

Additional file 5 (continued): Accession numbers and genome coordinates of ammonium transporter/ammonia permease genes included in the phylogenetic tree

Organism, gene abbreviation	GI number or GenBank accession number for DNA	Genome Region	GI number for mRNA	Clade
Aspergillus fumigatus, mepA	GI:71025132	(2822229..2823889)	GI:70997608	MEP γ
Aspergillus nidulans, meaA	GI:18693012	1478..>3330	N/A	MEP γ
Aspergillus nidulans, mepA	GI:18693014	1815..3504	N/A	MEP γ
Batrachochytrium dendrobatidis[B]	Supercontig 1	359666-361321	N/A	MEP γ
Candida albicans, MEP1	GI:68468731	(19689..21293)	GI:68468505	MEP γ
Candida albicans, MEP2	GI:68485553	54760..56202	GI:68485539	MEP γ
Candida albicans, 3*	GI:68484429	66692..68434	GI:68484407	N/A
Cladonia grayi, mep1a	JF833071	N/A	N/A	MEP α
Cladonia grayi, mep1b	JF833070	N/A	N/A	MEP α
Cladonia grayi, mep 2	JF833072	N/A	N/A	MEP γ
Cladonia grayi, mep3	JF833073	N/A	N/A	MEP γ
Coccidioides immitis, 1	GI:119185453	(3257148..3258822)	GI:119184212	MEP γ
Coccidioides immitis, 2	GI:119197146	2610936..2613096	GI:119193249	MEP γ
Coprinopsis cinerea okayama7#130, 1	GI:169866184	17197..18998	GI:169866180	MEP γ
Coprinopsis cinerea okayama7#130, 2	GI:169866153	6758..8559	GI:169866149	MEP γ
Coprinopsis cinerea okayama7#130, 3	GI:169844956	27679..29664	GI:169844550	MEP γ
Coprinopsis cinerea okayama7#130, 4	GI:169850323	(130995..133174)	GI:169850235	MEP γ
Cryptococcus neoformans var. *neoformans* JEC21, *AMT1*	GI:58271746	(585837..588247)	GI:58258402	MEP γ
Cryptococcus neoformans var. neoformans JEC21, *AMT2*	GI:58271747	(549599..551680)	GI:58259901	MEP γ
Fusarium graminearum PH-1, 1	GI:46562332	1934463..1936246	GI:46107473	MEP γ
Fusarium graminearum PH-1 ,2	GI:46562332	(6866192..6867738)	GI:46110424	MEP γ
Fusarium graminearum PH-1, 3	GI:46562332	1609324..1611001	GI:46107291	MEP γ
Fusarium graminearum PH-1, 4	GI:46562343	(765169..766784)	GI:46114441	MEP γ

Additional file 5 (continued): Accession numbers and genome coordinates of ammonium transporter/ammonia permease genes included in the phylogenetic tree

Organism, gene abbreviation	GI number or GenBank accession number for DNA	Genome Region	GI number for mRNA	Clade
Fusarium oxysporum f. sp. lycopersici 4286, 1	GI:144922572	(54000-55395)	N/A	MEP γ
Fusarium oxysporum f. sp. lycopersici 4286, 2	GI:144922350	57760-59300	N/A	MEP γ
Fusarium oxysporum f. sp. lycopersici 4286, 3	GI:144921882	33840-35337	N/A	MEP γ
Fusarium oxysporum f. sp. lycopersici 4286, 4	GI:144922346	(65941-67381)	N/A	MEP α
Fusarium oxysporum f. sp. lycopersici 4286, 5	GI:144922346	66000-67170	N/A	N/A
Gibberella moniliformis 7600, 1	GI:116139414	155125-156539	N/A	MEP γ
Gibberella moniliformis 7600, 2	GI:116139519	(424078-425576)	N/A	MEP γ
Gibberella moniliformis 7600, 3*	GI:116139519	~97480-~99000	N/A	N/A
Gibberella moniliformis 7600, 4	GI:116139410	132661-134281	N/A	MEP α
Gibberella moniliformis 7600 *	GI:116139361	59055-60699	N/A	N/A
Glomus intraradices, Gintamt1	N/A	N/A	GI:124516978	MEP γ
Hebeloma cylindrosporum, AMT1	N/A	N/A	GI:20501862	MEP γ
Hebeloma cylindrosporum, AMT2	N/A	N/A	GI:15042692	MEP γ
Hebeloma cylindrosporum, AMT3	N/A	N/A	GI:15042694	MEP γ
Laccaria bicolor, AMT1.1a	GI:170092085	(1141763..1143937	GI:170091535	MEP γ
Laccaria bicolor, AMT1.1b	GI:170115426	153134..155099	GI:17011531	MEP γ
Laccaria bicolor, AMT1.2	GI:170103048	(848838..851040)	GI:170103012	MEP γ
Laccaria bicolor, AMT2.1	GI:170086374	(798076..800048	GI:170085394	MEP γ
Laccaria bicolor, AMT2.2	GI:170110243	25357..26969	GI:170109925	MEP γ
Laccaria bicolor, AMT2.3a	GI:170112646	(379312..382864)	GI:170112632	MEP γ

Additional file 5 (continued): Accession numbers and genome coordinates of ammonium transporter/ammonia permease genes included in the phylogenetic tree

Organism, gene abbreviation	GI number or GenBank accession number for DNA	Genome Region	GI number for mRNA	Clade
Laccaria bicolor, AMT2.3b	GI:170112646	(368498..370363)	GI:170112630	MEP γ
Laccaria bicolor, AMT2.3c	GI:170115279	60031..61896	GI:170115107	MEP γ
Laurera megasperma, A	JF833075	N/A	N/A	MEP α
Laurera megasperma, B	JF833074	N/A	N/A	MEP α
Magnaporthe grisea 70-15, 1	GI:38104209	14334..16198	N/A	MEP γ
Magnaporthe grisea 70-15, 2	GI:38108755	(22223..23821)	N/A	MEP γ
Magnaporthe grisea 70-15, 3	GI:38101984	(9146..10992	N/A	MEP γ
Microbotryum violaceum, mepA	N/A	N/A	GI:5230671	MEP γ
Neurospora crassa, 1	GI:164426245	631321..633365	GI:164426202	MEP γ
Neurospora crassa, 2	GI:157070728	349091..350779	GI:85107332	MEP γ
Neurospora crassa, 3	GI:164427336	(221738..223494)	GI:85112787	MEP γ
Neurospora crassa, 4	GI:164426245	631321..633365	GI:164426202	MEP γ
Parmotrema submarginale (formerly Parmotrema michauxianum)	JF833076	N/A	N/A	MEP α
Penicillium chrysogenum, 1	GI:256353024	(8082430..8084162)	GI:255937472	MEP γ
Penicillium chrysogenum, 2	GI:256353024	(26853194..26855042)	GI:255948191	MEP γ
Penicillium chrysogenum, 3	GI:211588786	(2625848..2627524)	GI:255954772	MEP γ
*Penicillium chrysogenum**	GI:256353024	(21836862..21838538)	GI:255954772	N/A
Penicillium marneffei, 1	GI:212544102	(1035187..1037338)	GI:212542532	MEP γ
Penicillium marneffei, 2	GI:212536833	(165518..167568)	GI:212534103	MEP γ
Penicillium marneffei, 3	GI:212530903	3680024..3682106	GI:212528861	MEP γ
Penicillium marneffei, 4	GI:212530903	(4869932..4871615)	GI:212529771	MEP α
Penicillium marneffei, 5	GI:212534004	212731..214581	GI:212531034	MEP γ
Pyrenophora tritici-repentis, 1	GI:189192591	2218689..2220719	GI:189189627	MEP γ
Pyrenophora tritici-repentis, 2	GI:189206496	(1468324..1469966)	GI:189206414	MEP γ

Additional file 5 (continued): Accession numbers and genome coordinates of ammonium transporter/ammonia permease genes included in the phylogenetic tree

Organism, gene abbreviation	GI number or GenBank accession number for DNA	Genome Region	GI number for mRNA	Clade
Pyrenophora tritici-repentis, 3	GI:189192591	5135420..5137396	GI:189191563	MEP γ
Pyrenophora tritici-repentis, 4	GI:189196879	1673492..1675071	GI:189196725	MEP γ
Pyrenophora tritici-repentis, 5	GI:189198580	1673492..1675071	GI:189197910	MEP γ
Pyrenula cruenta	JF833077	N/A	N/A	MEP α
Ramalina sp., A	JF833078	N/A	N/A	MEP α
Ramalina sp., B	JF833079	N/A	N/A	MEP α
Rhizopus oryzae, 1[B]	Supercontig 3	(407915-410079)	N/A	MEP γ
Rhizopus oryzae, 2[B]	Supercontig 9	(212386-214521)	N/A	MEP γ
Rhizopus oryzae, 3[B]	Supercontig 10	1263026-1264373	N/A	MEP γ
Rhizopus oryzae, 4[B]	Supercontig 10	1726208-1727707	N/A	MEP γ
Rhizopus oryzae, 5[B]	Supercontig 4	(668186-669408)	N/A	MEP γ
Saccharomyces cerevisiae, MEP1	GI:162949218	(731454..732932)	N/A	MEP γ
Saccharomyces cerevisiae, MEP2	GI:117937805	357455..358954	N/A	MEP γ
Saccharomyces cerevisiae, MEP3	GI:50593503	(810980..812449)	N/A	MEP γ
Schizosaccaromyces pombe, amt1	GI:63054406	2069806..2071299	GI:68037248	MEP γ
Schizosaccaromyces pombe, amt2	GI:162312575	1731627..1733165	GI:67999505	MEP γ
Schizosaccharomyces pombe, amt3	GI:162312575	(2924893..2926446)	GI:68000034	MEP γ
Sclerotinia sclerotiorum, 1	GI:156053526	(748453..750262)	GI:156052866	MEP γ
Sclerotinia sclerotiorum, 2	GI:156058194	(1380869..1382769)	GI:156057540	MEP γ
Sclerotinia sclerotiorum, 3	GI:156056511	145159..146698	GI:156055065	MEP γ
Stereocaulon tennesseense	JF833080	N/A	N/A	MEP α
Talaromyces stipitatus, 1	GI:242771011	(4296843..4298562)	GI:242768291	MEP γ
Talaromyces stipitatus, 2	GI:242797504	(198426..200031)	GI:218717854	MEP γ
Talaromyces stipitatus, 3	GI:242771011	(2855574..2857925)	GI:242765292	MEP γ
Talaromyces stipitatus, 4	GI:242771011	1387342..1389023	GI:242762291	MEP α

Additional file 5 (continued): Accession numbers and genome coordinates of ammonium transporter/ammonia permease genes included in the phylogenetic tree

Organism, gene abbreviation	GI number or GenBank accession number for DNA	Genome Region	GI number for mRNA	Clade
Talaromyces stipitatua, 5	GI:218721414	259064..260882	GI:242771613	MEP γ
Trypethelium virens	JF833081	N/A	N/A	MEP α
Tuber borchii, *AMT1*	N/A	N/A	GI:17221149	MEP γ
Usnea mutabilis, A	JF833082	N/A	N/A	MEP α
Usnea mutabilis, B	JF833083	N/A	N/A	MEP α
Ustilago maydis 521, *mep1*	GI:49083521	(110635..112196)	GI:71023612	MEP γ
Ustilago maydis 521, *ump2*	GI:49083411	45715..47301	GI:71020878	MEP γ
**Slime molds (Amoebozoa)**				
Dictyostelium discoideum AX4, *amtC*	GI:269316041	1034624-1036114	GI:31323876	grade
**Chromalveolates**				
**Stramenopiles (Heterokonts)**				
Phytophthora infestans T30-4, 1[J]	>gnl|WGS:AATU|cont1.466	35000-39000	N/A	MEP β
Phytophthora infestans T30-4, 2[J]	>gnl|WGS:AATU|cont1.2760	2830-4800	N/A	MEP β
Phytophthora infestans T30-4, 3[J]	>gnl|WGS:AATU|cont1.8908	1-700	N/A	MEP β
**Excavates**				
**Euglenozoa**				
Trypanosoma cruzi strain CL Brener	GI:70876681	73022-74566	GI:71421943	grade
**Heterolobosea**				
Naegleria gruberi, *amtA*[J]	scaffold_4	165684-167083	N/A	grade

Additional file 5 (continued): Accession numbers and genome coordinates of ammonium transporter/ammonia permease genes included in the phylogenetic tree

Organism, gene abbreviation	GI number or GenBank accession number for DNA	Genome Region	GI number for mRNA	Clade
**PROKARYOTES**				
**Archaea**				
Archaeoglobus fulgidus, *amt-1*	GI:11497621	879899..881074	N/A	MEP α
Archaeoglobus fulgidus, *amt-3*	GI:11497621	1570632..1571798	N/A	MEP α
Caldivirga maquilingensis IC-167	GI:159040592	715687..717117	N/A	MEP α
Ferroplasma acidarmanus	GI:126008996	221620..222921	N/A	MEP α
Methanosarcina acetivorans C2A, *amtB* (1)	GI:20088899	4815556..4816764	N/A	grade
Methanosarcina barkeri Meth_1922, 2	GI:23052039	(985..2202)	N/A	grade
Methanosarcina barkeri Meth_1922, 1	GI:23052039	(2418..3617)	N/A	MEP β
Methanosarcina mazei Go1 1	GI:21226102	881714..882913	N/A	MEP β
Methanosphaera stadtmanae DSM 3091	GI:84488831	774206-775429	N/A	MEP α
Methanothermobacter thermautotrophicus str. Delta H, 1	GI:15678031	588943-590166	N/A	MEP α
Methanothermobacter thermautotrophicus str. Delta H, 2	GI:15678031	590781-592019	N/A	MEP α
Picrophilus torridus DSM 9790	GI:48477072	1460613-1461911	N/A	MEP α
Sulfolobus tokodaii str. 7	GI:24473558	643486-645042	N/A	MEP α
Sulfolobus solfataricus	GI:15896971	914084..915613	N/A	MEP α
**Bacteria**				
Acidimicrobium ferrooxidans DSM 10331	GI:256370824	2108845-2110290	N/A	MEP α
Acidithiobacillus caldus ATCC 51756, *amt-1*	ACVD01000125.1	109..834	N/A	MEP α
Acidithiobacillus caldus ATCC 51756, *amt-*	ACVD01000080.1	19056..20363	N/A	N/A

Additional file 5 (continued): Accession numbers and genome coordinates of ammonium transporter/ammonia permease genes included in the phylogenetic tree

Organism, gene abbreviation	GI number or GenBank accession number for DNA	Genome Region	GI number for mRNA	Clade
2*				
Acidithiobacillus ferrooxidans ATCC 53993 *amt-2*	GI:198282148	2491440..2492756	N/A	MEP β
Acidithiobacillus ferrooxidans ATCC 53993, *amt-1*	GI:198282148	2497431-2498735	N/A	MEP α
Acidithiobacillus ferrooxidans ATCC 23270 *amt-2*	GI:218665024	2589616-2590932	N/A	MEP β
Acidithiobacillus ferrooxidans ATCC 23270 *amt-1*	GI:218665024	2595607-2596911	N/A	MEP α
Agrobacterium tumefaciens str. C58 (Cereon), a*mtB*	GI:159184118	2757775..2758980	N/A	MEP β
Aquifex aeolicus VF5	GI:15282445	69169-70440	N/A	MEP α
Azoarcus sp. BH72, *amtB%*	GI:19851906	N/A	N/A	MEP β
Azorhizobium caulinodans, amtB	GI:2980822	N/A	N/A	MEP β
Azospirillum brasilense, amtB	GI:3136067	N/A	N/A	MEP β
Azotobacter vinelandii DJ, *amtB*	GI:226942170	4846081-4847397	N/A	MEP β
Azotobacter vinelandii, amtB	GI:29359385	165528..166787	N/A	MEP β
Bacillus cereus ATCC 14579	GI:30018278	(1135908..1137140)	N/A	MEP β
Bacillus subtilis subsp. subtilis str. 168 *amtB*	GI:255767013	3756790..3758004	N/A	MEP β
Bifidobacterium longum DJO10A, *amtB*	GI:29251702	(91351..92583)	N/A	MEP β
Bifidobacterium longum NCC2705, *amtP*	GI:58036264	(1382167..1383462)	N/A	MEP β
Bordetella bronchiseptica RB50, *amtB*	GI:33575370	(93085..94323)	N/A	MEP β
Bordetella parapertussis	GI:33573592	(10300..11538)	N/A	MEP β
Bradyrhizobium japonicum USDA 110	GI:27375111	652211..653227	N/A	N/A

Additional file 5 (continued): Accession numbers and genome coordinates of ammonium transporter/ammonia permease genes included in the phylogenetic tree

Organism, gene abbreviation	GI number or GenBank accession number for DNA	Genome Region	GI number for mRNA	Clade
chromosome*				
Bradyrhizobium japonicum USDA 110 chromosome*	GI:27375111	652842..653513	N/A	N/A
Bradyrhizobium japonicum USDA 110 chromosome, amtB	GI:27375111	655678..657210	N/A	MEP β
Brevibacterium linens BL2	GI:62422953	(16634..17854)	N/A	MEP β
Brevibacterium linens=Burkholderia vietnamensis G4 (1)	GI:134137285	(3282231..3283730)	N/A	MEP β
Brucella melitensis	GI:17986284	161196..162497	N/A	MEP β
Brucella suis 1330, *amt*	GI:56968325	(1831408..1832709)	N/A	MEP β
Brucella suis ATCC 23445 chromosome I	GI:163673000	1647375-1648676	N/A	MEP β
Burkholderia fungorum Bcep_273, 1	GI:28873745	45938..47218	N/A	MEP β
Burkholderia sp. 383 (=*Burkholderia cepacia* R-18194) (1)	GI:77965403	(3341542..3343056)	N/A	MEP β
Carboxydothermus hydrogenoformans Z-2901	GI:77994731	62220-63536	N/A	MEP β
Caulobacter crescentus CB15	GI:16124256	(1492580..1494112)	N/A	MEP β
Chlorobium chlorochromatii CaD3 (=*Chlorochromatium aggregatum*)	GI:78170183	2533520..2534854	N/A	MEP β
Chlorobium tepidum TLS, *amt-3*	GI:21672841	128241..12957	N/A	MEP β
Chromobacterium violaceum ATCC 12472, *amtB*	GI:34495455	4329876..4331186	N/A	MEP β
Clostridium acetobutylicum, *nrgA*	GI:15893970	(787791..789008)	N/A	MEP β
Corynebacterium efficiens YS-314, 2	GI:25028257	(1790505..1791824)	N/A	MEP β
Corynebacterium efficiens YS-314, *amtP*	GI:25028524	(2073802..2075127)	N/A	MEP β

Additional file 5 (continued): Accession numbers and genome coordinates of ammonium transporter/ammonia permease genes included in the phylogenetic tree

Organism, gene abbreviation	GI number or GenBank accession number for DNA	Genome Region	GI number for mRNA	Clade
Corynebacterium glutamicum ATCC 13032, 2	GI:58036263	1675265-1676623	N/A	MEP β
Corynebacterium glutamicum ATCC 13032, 1	GI:58036263	(2172151..2173467)	N/A	MEP β
Cyanothece sp. PCC 7822	GI:196258658	210450..211856	N/A	MEP β
Cytophaga hutchinsonii, amtB, 1	GI:110636427	(3786660..3787988)	N/A	N/A
Dechloromonas aromatica RCB, 1	GI:71845263	62898..64397	N/A	MEP β
Deinococcus radiodurans	GI:15805720	708354..709673	N/A	MEP β
Desulfitobacterium hafniense	GI:30471518	19330..20676	N/A	MEP β
Desulfococcus oleovorans Hxd3, 2	GI:158520017	(633089-634375)	N/A	MEP α
Desulfococcus oleovorans Hxd3, 1	GI:158520017	(634397-635680)	N/A	MEP β
Desulfovibrio desulfuricans G20, 1	GI:28877455	62208..63413	N/A	MEP β
Desulfovibrio desulfuricans, 2	GI:78217452	(2328899..2330104)	N/A	MEP β
Desulfuromonas acetoxidans, DSM 684 ctg55, 1	GI:95133343	70185..71510	N/A	MEP β
Enterococcus faecium	GI:22992526	3209..4423	N/A	grade
Escherichia coli B str. REL606*	GI:253972022	445033-446319	N/A	N/A
Escherichia coli str. K-12 substr. MG1655	GI:49175990	472256..473473	N/A	MEP β
Geobacter metallireducens GS-15, 1	GI:23054753	7603..9069	N/A	MEP β
Geobacter sulfurreducens PCA, 1	GI:39995111	1009771..1011240	N/A	MEP β
Geobacter sulfurreducens PCA, 2	GI:39995111	(1324618..1325838)	N/A	grade
*Gloeobacter violaceus**	GI:37519569	(2798589..2799443)	N/A	N/A
Gloeobacter violaceus	GI:37519569	3253283..3254719	N/A	MEP β
*Gluconacetobacter diazotrophicus, amtB2**	GI:209542188	1454476..1455855	N/A	N/A
Gluconacetobacter diazotrophicus, amtB1	GI:209542188	669752..671143	N/A	MEP β

Additional file 5 (continued): Accession numbers and genome coordinates of ammonium transporter/ammonia permease genes included in the phylogenetic tree

Organism, gene abbreviation	GI number or GenBank accession number for DNA	Genome Region	GI number for mRNA	Clade
Haemophilus somnus 129PT, *amtB*	GI:28301892	19208..20506	N/A	MEP β
Lactobacillus casei, str. Zhang, *amtB*	GI:300437532	(592027-593343)	N/A	MEP β
Lactobacillus plantarum WCFS1, *amtB*	GI:28269805	(318549..319865)	N/A	MEP β
Lactococcus lactis subsp cremoris MG1363, *amtB*	GI:124491690	876626..877867	N/A	MEP β
Lactococcus lactis subsp. lactis Il1403, *amtB*	GI:13400022	(1635861..1637102)	N/A	MEP β
Leptospira interrogans serovar lai str. 56601, *amtB**	GI:294827553	(3577147..3579492)	N/A	N/A
Leptospirillum rubarum LeptoII_Scaffold_8241	GI:124516098	100910-102217	N/A	MEP α
Leuconostoc mesenteroides subsp. mesenteroides ATCC 8293	GI:28876869	51528..52724	N/A	MEP β
Listeria innocua	GI:16414035	2144..3362	N/A	MEP β
Listeria monocytogenes EGD-e	GI:1641081	138688..139906	N/A	MEP β
Magnetococcus sp. MC-1	GI:23000293	19671..20981	N/A	MEP β
Mesorhizobium loti, amtB	GI:57165207	(3387960..3389315)	N/A	MEP β
*Methanocaldococcus jannaschii, amtB**	GI:15668172	(57588..58763)	N/A	N/A
Methylobacillus flagellatus KT, 1	GI:91774356	2693851-2695236	N/A	MEP β
Methylobacillus flagellatus KT, 2	GI:91774356	442257-443561	N/A	MEP β
Mycobacterium tuberculosis H37Rv, *amt*	GI:41352756	(112036..113469)	N/A	MEP β
Nautilia profundicola AmH, amt (1)	GI:224372070	185564-186844	N/A	MEP α
Nautilia profundicola AmH (duplicate)*	GI:223588326	185564-186844	N/A	N/A
Neisseria meningitidis MC58, *amtB*	GI:7735869	(645072..646376)	N/A	MEP β
Neisseria meningitidis Z2491, *amtB*	GI:15793034	(801446..802750)	N/A	MEP β

Additional file 5 (continued): Accession numbers and genome coordinates of ammonium transporter/ammonia permease genes included in the phylogenetic tree

Organism, gene abbreviation	GI number or GenBank accession number for DNA	Genome Region	GI number for mRNA	Clade
Nostoc punctiforme PCC 73102 (1)	GI:186680550	4091213-4092625	N/A	MEP β
Nostoc punctiforme PCC 73102*	GI:30581866	99651..101111	N/A	N/A
Nostoc punctiforme PCC 73102*	GI:30581855	(31482..32906)	N/A	N/A
Nostoc sp. PCC 7120*	GI:17227497	1158426..1159982	N/A	N/A
Nostoc sp. PCC 7120, 2	GI:17227497	1160381..1161793	N/A	MEP β
Novosphingobium aromaticivorans, DSM 12444	GI:87198026	2449396..2450709	N/A	MEP β
Novosphingobium aromaticivorans, DSM 12444*	GI:87198026	733046..734404	N/A	N/A
Oenococcus oeni MC	GI:29378884	(34354..35700)	N/A	MEP β
Photorhabdus luminescens subsp. laumondii TTO1, *amtB*	GI:37524032	(4529787..4531106)	N/A	MEP β
Pseudomonas aeruginosa PA01, *amtB*	GI:110645304	(5951115..5952443)	N/A	MEP β
Pseudomonas fluorescens PfO-1, 1	GI:253992019	(6170031..6171368)	N/A	MEP β
Pseudomonas putida KT2440, *amtB*	GI:26986745	(5966352..5967683)	N/A	MEP β
Pseudomonas stutzeri, *amtB2*	GI:19033132	3281..4534	N/A	grade
Pseudomonas syringae pv. syringae B728a, 1	GI:66043271	(200642..201979)	N/A	MEP β
Pseudomonas syringae pv. syringae B728a, 2	GI:66043271	2643672..2644997	N/A	MEP β
Pseudomonas syringae pv. tomato str. DC3000, *amt-1*	GI:28867243	240728..242065	N/A	MEP β
Pseudomonas syringae pv. tomato str. DC3000, *amt-2*	GI:28867243	2856588..2857913	N/A	MEP β
Ralstonia eutropha JMP134, 1	GI:72117119	324070..325611	N/A	MEP β

Additional file 5 (continued): Accession numbers and genome coordinates of ammonium transporter/ammonia permease genes included in the phylogenetic tree

Organism, gene abbreviation	GI number or GenBank accession number for DNA	Genome Region	GI number for mRNA	Clade
Ralstonia metallidurans, amtB (=*Cupriavidus metallidurans* CH34)	GI:93352797	256874..258406	N/A	MEP β
Ralstonia solanacearum, amtB	GI:17544719	374899..376209	N/A	MEP β
Rhizobium etli, amtB	GI:6599354	787..2211	N/A	MEP β
Rhodobacter sphaeroides, amtB	GI:77461965	(2636500..2637852	N/A	MEP β
Rhodopirullela baltica SH1, 2	GI:32397972	137662..139158	N/A	grade
Rhodopseudomonas palustris, amtB1	GI:39652705	300666..301997	N/A	MEP β
Rhodopseudomonas palustris, amtB2	GI:39652705	302683..304125	N/A	MEP β
Rhodospirillum rubrum ATCC 11170, 1	GI:83574254	(1330513..1331808)	N/A	MEP β
Saccharophagus degradans (=*Microbulbifer degradans*) *amtB1*	GI:89949249	(2643236..2644531)	N/A	MEP β
Salmonella enterica subsp. enterica serovar Typhi, *amtB*	GI:16501496	262755..264041	N/A	MEP β
Shigella flexneri 2a str. 301, *amtB*	GI:24111450	408059..409345	N/A	MEP β
Sinorhizobium meliloti, amtB	GI:15963753	3471607..3472962	N/A	MEP β
Staphylococcus aureus subsp. aureus Mu50, *nrgA*	GI:57634611	(2162727..2163977)	GI:83574254	MEP β
Staphylococcus aureus subsp. aureus MW2, *nrgA*	GI:47118312	(2114899..2116149)	N/A	MEP β
Staphylococcus epidermidis ATCC 12228	GI:27466918	(1696434..1697684)	N/A	MEP β
Streptococcus mutans UA159, *nrgA*	GI:24378532	1575845..1577080)	N/A	MEP β
Streptomyces avermitilis MA-4680, *amtB1*	GI:162960844	(3248960..3250300)	N/A	MEP β
Streptomyces avermitilis MA-4680, *amtB2*	GI:162960844	(6766506..6767816)	N/A	MEP β
Streptomyces coelicolor	GI:24413886	105031..106377	N/A	MEP β
Sulfurihydrogenibium sp. YO3AOP1	GI:188931022	(55106..56395)	N/A	MEP α

Additional file 5 (continued): Accession numbers and genome coordinates of ammonium transporter/ammonia permease genes included in the phylogenetic tree

Organism, gene abbreviation	GI number or GenBank accession number for DNA	Genome Region	GI number for mRNA	Clade
Synechococcus elongatus PCC 7942, 1	GI:81167692	2347614..2349026	N/A	MEP β
Synechocystis sp. PCC 6803, *amt2*	GI:16329170	(400720..402048)	N/A	grade
Thermobifida fusca, *amtB*	GI:72160406	764337..765656	N/A	MEP β
Ureaplasma urealyticum, *amt-1*	GI:13357558	260648..262180	N/A	grade
Ureaplasma urealyticum, *amt-2*	GI:13357558	262345..263910	N/A	grade
Wolinella succinogenes, *amtB*	GI:34482500	(51759..53072)	N/A	MEP α
Xanthomonas campestris pv. campestris str. ATCC 33913, *amtB*	GI:21166373	243559..245034	N/A	MEP β
Xanthomonas citri, *amtB*	GI:5917780	N/A	N/A	MEP β
Xylella fastidiosa 9a5c	GI:57014152	1759686..1761164	N/A	MEP β
Xylella fastidiosa Dixon	GI:22994217	20672..22057	N/A	MEP β
Xylella fastidiosa Temecula1, *amtB*	GI:28197945	(1222493..1223947)	N/A	MEP β
Yersinia pestis CO92, *amtB*	GI:16120353	(3501231..3502526)	N/A	MEP β

%= duplicate sequence in the analysis, * = excluded or omitted from the analysis, [J] = data from http://www.jgi.doe.gov/; [B]=data from http://www.broadinstitute.org/;
