# Supplementary material for: High-throughput genome sequencing of lichenizing fungi to assess gene loss in the ammonium transporter/ammonia permease gene family
Source: BMC Genomics. 2013 Apr 4;14:225. doi: 10.1186/1471-2164-14-225 (PMC3663718; doi:10.1186/1471-2164-14-225)
Supplement: Additional file 6 — Phylogenetic placement of ammonium transporters/ammonia permeases from eight lichen genomes. Maximum likelihood analysis of 300 ammonium transporter/ammonia permease genes details the phylogenetic placement of ammonium transporter/ammonia permease genes in the well-supported predominantly prokaryotic clade (MEP) in which eukaryotic lineages demonstrate horizontal gene transfer. [file 1471-2164-14-225-S6.pdf]

Additional file 6 - Phylogenetic placement of ammonium transporters/ammonia permeases from eight lichen genomes. Maximum likelihood analysis of 300 ammonium transporter/ammonia permease genes details the phylogenetic placement of ammonium transporter/ammonia permease genes in the well-supported predominantly prokaryotic clade (MEP) in which eukaryotic lineages demonstrate horizontal gene transfer. MEP=methylammonium permease clade; MEP grade = prokaryotic ammonium transporter/ammonia. Thickened branches show bootstrap support of ≥70%.

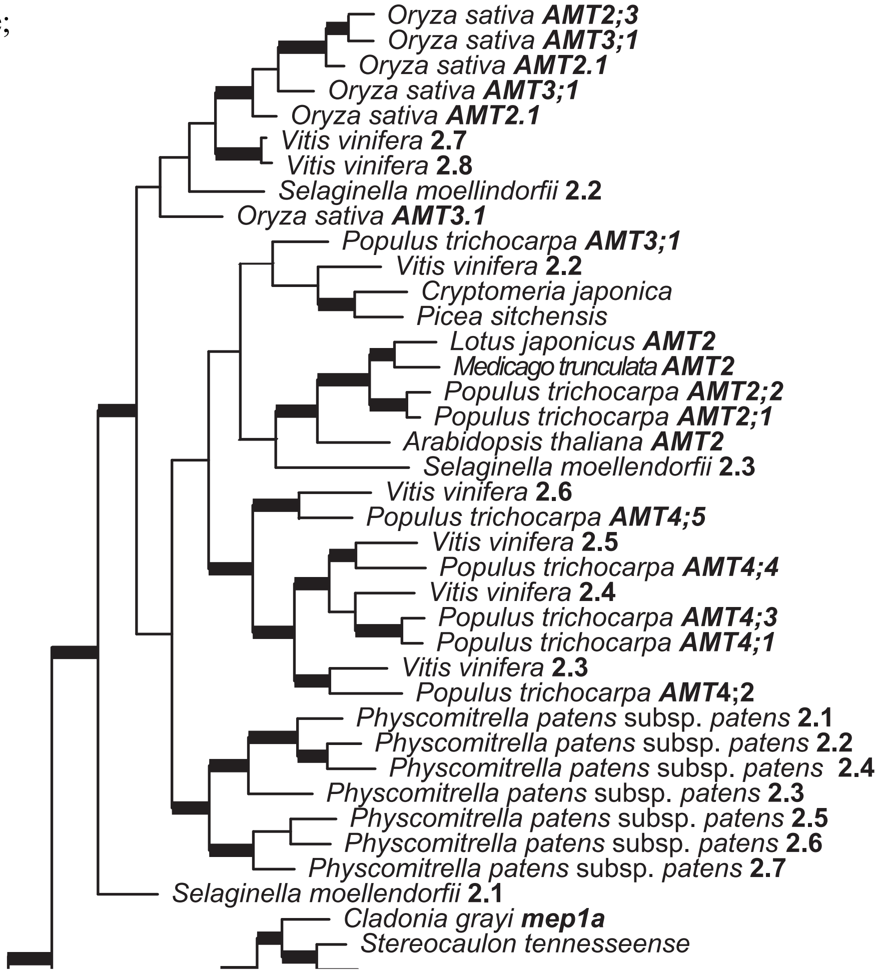

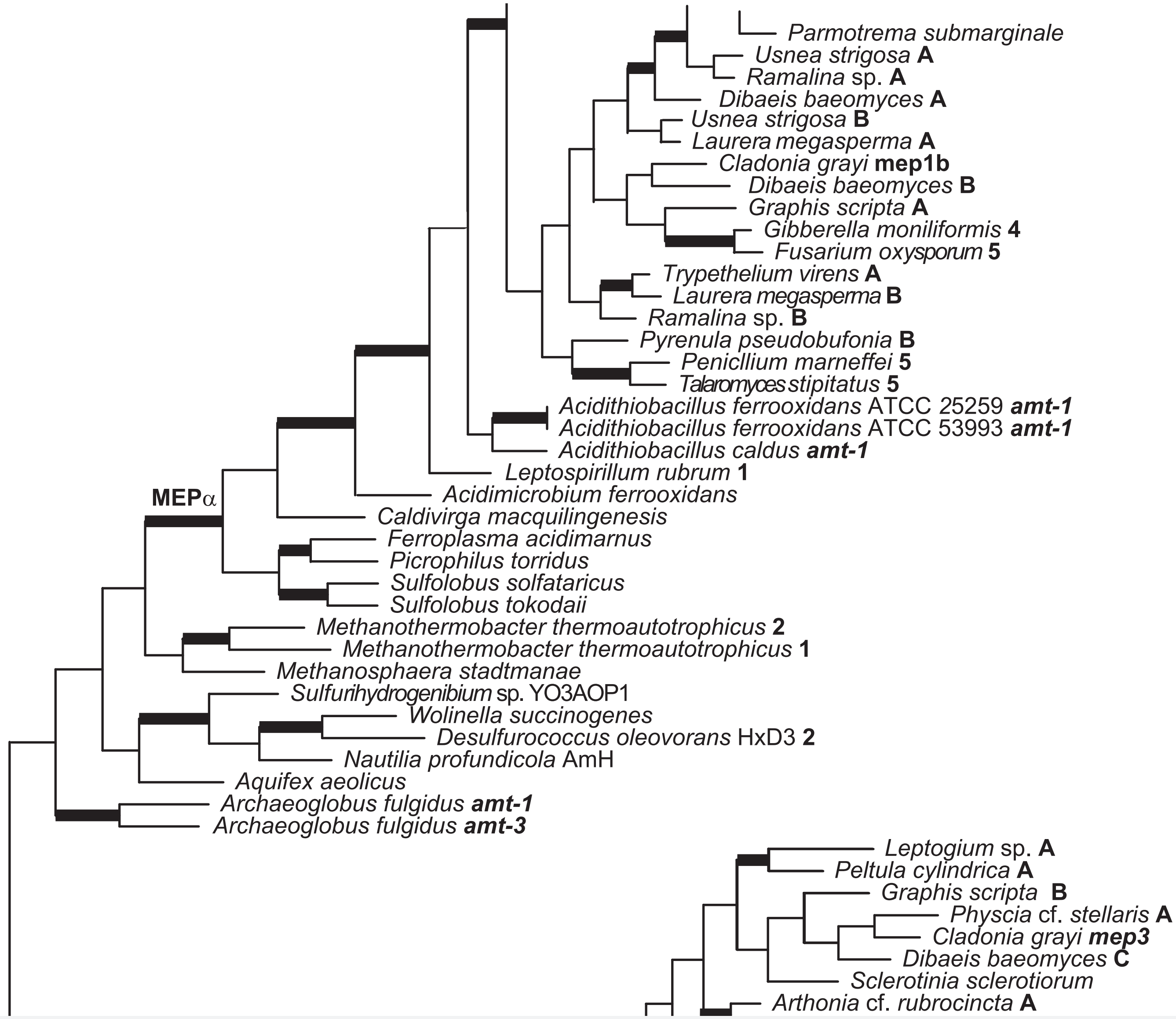

MEPα
Parmotrema submarginale
Usnea strigosa A
Ramalina sp. A
Dibaeis baeomyces A
Usnea strigosa B
Laurera megasperma A
Cladonia grayi mep1b
Dibaeis baeomyces B
Graphis scripta A
Gibberella moniliformis 4
Fusarium oxysporum 5
Trypethelium virens A
Laurera megasperma B
Ramalina sp. B
Pyrenula pseudobufonia B
Penicllium marneffei 5
Talaromyces stipitatus 5
Acidithiobacillus ferrooxidans ATCC 25259 amt-1
Acidithiobacillus ferrooxidans ATCC 53993 amt-1
Acidithiobacillus caldus amt-1
Leptospirillum rubrum 1
Acidimicrobium ferrooxidans
Caldivirga macquilingenesis
Ferroplasma acidimarnus
Picrophilus torridus
Sulfolobus solfataricus
Sulfolobus tokodaii
Methanothermobacter thermoautotrophicus 2
Methanothermobacter thermoautotrophicus 1
Methanosphaera stadtmanae
Sulfurihydrogenibium sp. YO3AOP1
Wolinella succinogenes
Desulfurococcus oleovorans HxD3 2
Nautilia profundicola AmH
Aquifex aeolicus
Archaeoglobus fulgidus amt-1
Archaeoglobus fulgidus amt-3
Leptogium sp. A
Peltula cylindrica A
Graphis scripta B
Physcia cf. stellaris A
Cladonia grayi mep3
Dibaeis baeomyces C
Sclerotinia sclerotiorum
Arthonia cf. rubrocincta A

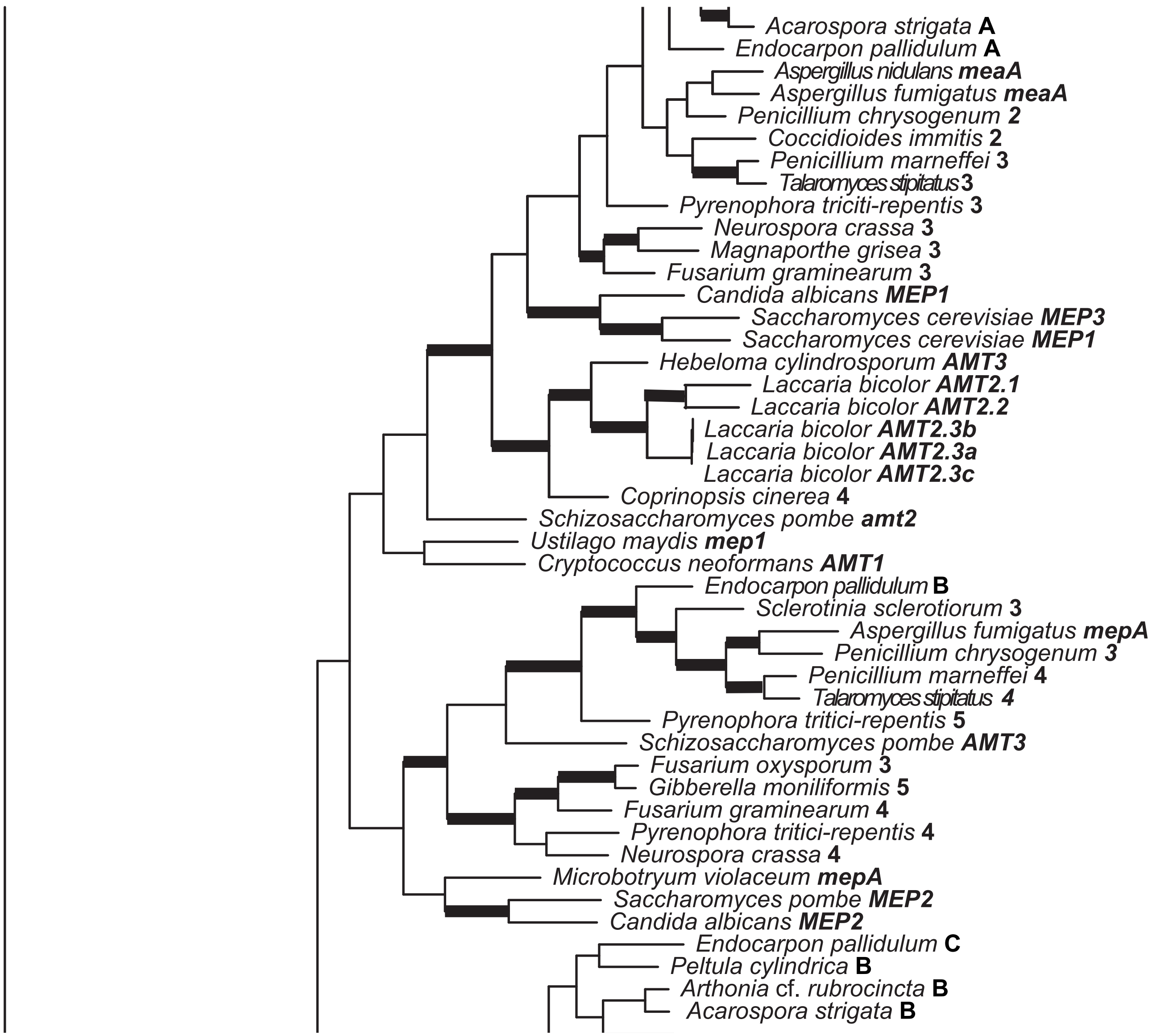

Acarospora strigata A
Endocarpon pallidulum A
Aspergillus nidulans meaA
Aspergillus fumigatus meaA
Penicillium chrysogenum 2
Coccidioides immitis 2
Penicillium marneffei 3
Talaromyces stipitatus 3
Pyrenophora triciti-repentis 3
Neurospora crassa 3
Magnaporthe grisea 3
Fusarium graminearum 3
Candida albicans MEP1
Saccharomyces cerevisiae MEP3
Saccharomyces cerevisiae MEP1
Hebeloma cylindrosporum AMT3
Laccaria bicolor AMT2.1
Laccaria bicolor AMT2.2
Laccaria bicolor AMT2.3b
Laccaria bicolor AMT2.3a
Laccaria bicolor AMT2.3c
Coprinopsis cinerea 4
Schizosaccharomyces pombe amt2
Ustilago maydis mep1
Cryptococcus neoformans AMT1
Endocarpon pallidulum B
Sclerotinia sclerotiorum 3
Aspergillus fumigatus mepA
Penicillium chrysogenum 3
Penicillium marneffei 4
Talaromyces stipitatus 4
Pyrenophora tritici-repentis 5
Schizosaccharomyces pombe AMT3
Fusarium oxysporum 3
Gibberella moniliformis 5
Fusarium graminearum 4
Pyrenophora tritici-repentis 4
Neurospora crassa 4
Microbotryum violaceum mepA
Saccharomyces pombe MEP2
Candida albicans MEP2
Endocarpon pallidulum C
Peltula cylindrica B
Arthonia cf. rubrocincta B
Acarospora strigata B

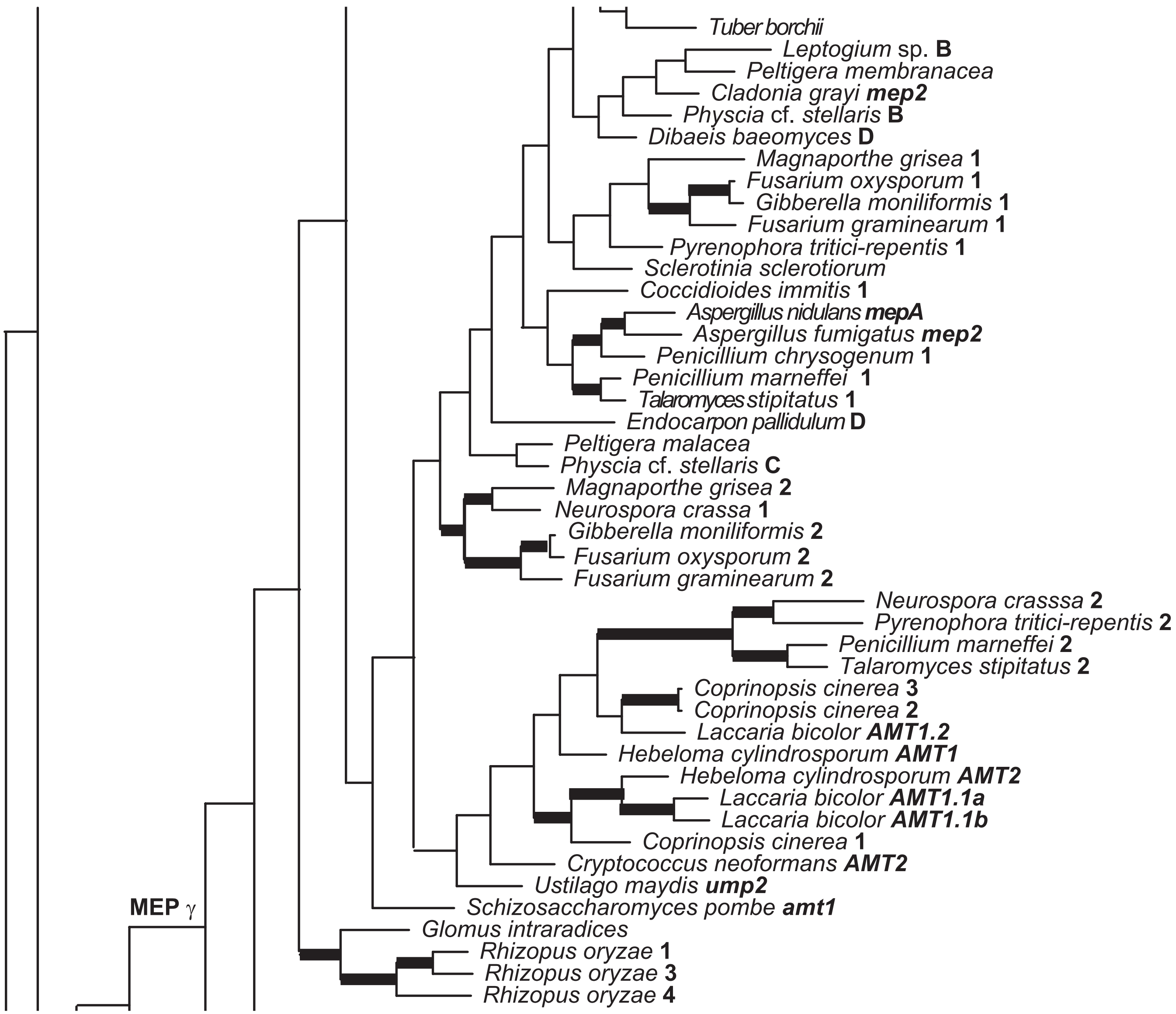

Tuber borchii
Leptogium sp. B
Peltigera membranacea
Cladonia grayi mep2
Physcia cf. stellaris B
Dibaeis baeomyces D
Magnaporthe grisea 1
Fusarium oxysporum 1
Gibberella moniliformis 1
Fusarium graminearum 1
Pyrenophora tritici-repentis 1
Sclerotinia sclerotiorum
Coccidioides immitis 1
Aspergillus nidulans mepA
Aspergillus fumigatus mep2
Penicillium chrysogenum 1
Penicillium marneffei 1
Talaromyces stipitatus 1
Endocarpon pallidulum D
Peltigera malacea
Physcia cf. stellaris C
Magnaporthe grisea 2
Neurospora crassa 1
Gibberella moniliformis 2
Fusarium oxysporum 2
Fusarium graminearum 2
Neurospora crasssa 2
Pyrenophora tritici-repentis 2
Penicillium marneffei 2
Talaromyces stipitatus 2
Coprinopsis cinerea 3
Coprinopsis cinerea 2
Laccaria bicolor AMT1.2
Hebeloma cylindrosporum AMT1
Hebeloma cylindrosporum AMT2
Laccaria bicolor AMT1.1a
Laccaria bicolor AMT1.1b
Coprinopsis cinerea 1
Cryptococcus neoformans AMT2
Ustilago maydis ump2
Schizosaccharomyces pombe amt1
MEP γ
Glomus intraradices
Rhizopus oryzae 1
Rhizopus oryzae 3
Rhizopus oryzae 4

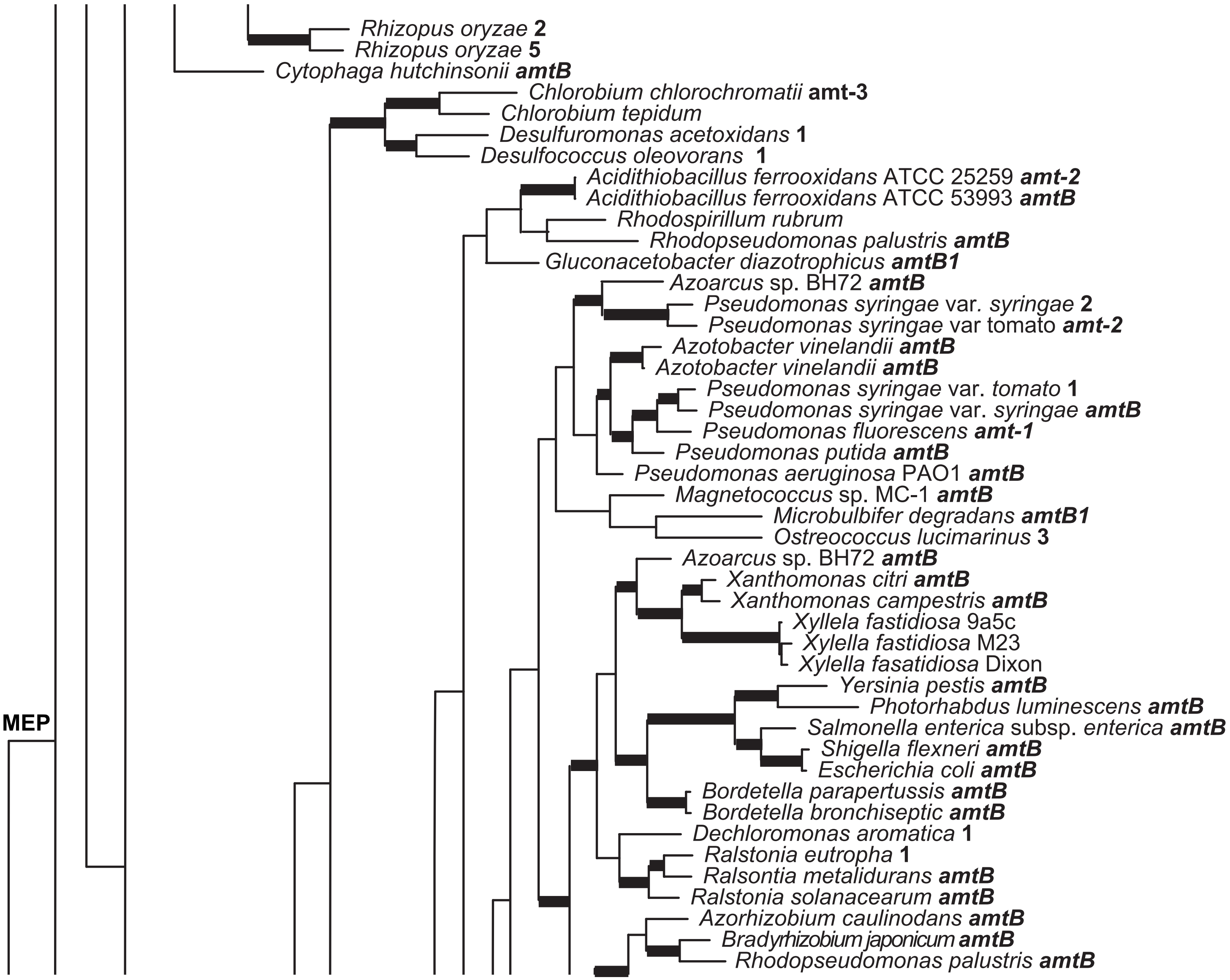
Rhizopus oryzae 2
Rhizopus oryzae 5
Cytophaga hutchinsonii amtB
Chlorobium chlorochromatii amt-3
Chlorobium tepidum
Desulfuromonas acetoxidans 1
Desulfococcus oleovorans 1
Acidithiobacillus ferrooxidans ATCC 25259 amt-2
Acidithiobacillus ferrooxidans ATCC 53993 amtB
Rhodospirillum rubrum
Rhodopseudomonas palustris amtB
Gluconacetobacter diazotrophicus amtB1
Azoarcus sp. BH72 amtB
Pseudomonas syringae var. syringae 2
Pseudomonas syringae var tomato amt-2
Azotobacter vinelandii amtB
Azotobacter vinelandii amtB
Pseudomonas syringae var. tomato 1
Pseudomonas syringae var. syringae amtB
Pseudomonas fluorescens amt-1
Pseudomonas putida amtB
Pseudomonas aeruginosa PAO1 amtB
Magnetococcus sp. MC-1 amtB
Microbulbifer degradans amtB1
Ostreococcus lucimarinus 3
Azoarcus sp. BH72 amtB
Xanthomonas citri amtB
Xanthomonas campestris amtB
Xyllela fastidiosa 9a5c
Xylella fastidiosa M23
Xylella fasatidiosa Dixon
Yersinia pestis amtB
Photorhabdus luminescens amtB
Salmonella enterica subsp. enterica amtB
Shigella flexneri amtB
Escherichia coli amtB
Bordetella parapertussis amtB
Bordetella bronchiseptic amtB
Dechloromonas aromatica 1
Ralstonia eutropha 1
Ralsontia metalidurans amtB
Ralstonia solanacearum amtB
Azorhizobium caulinodans amtB
Bradyrhizobium japonicum amtB
Rhodopseudomonas palustris amtB
MEP

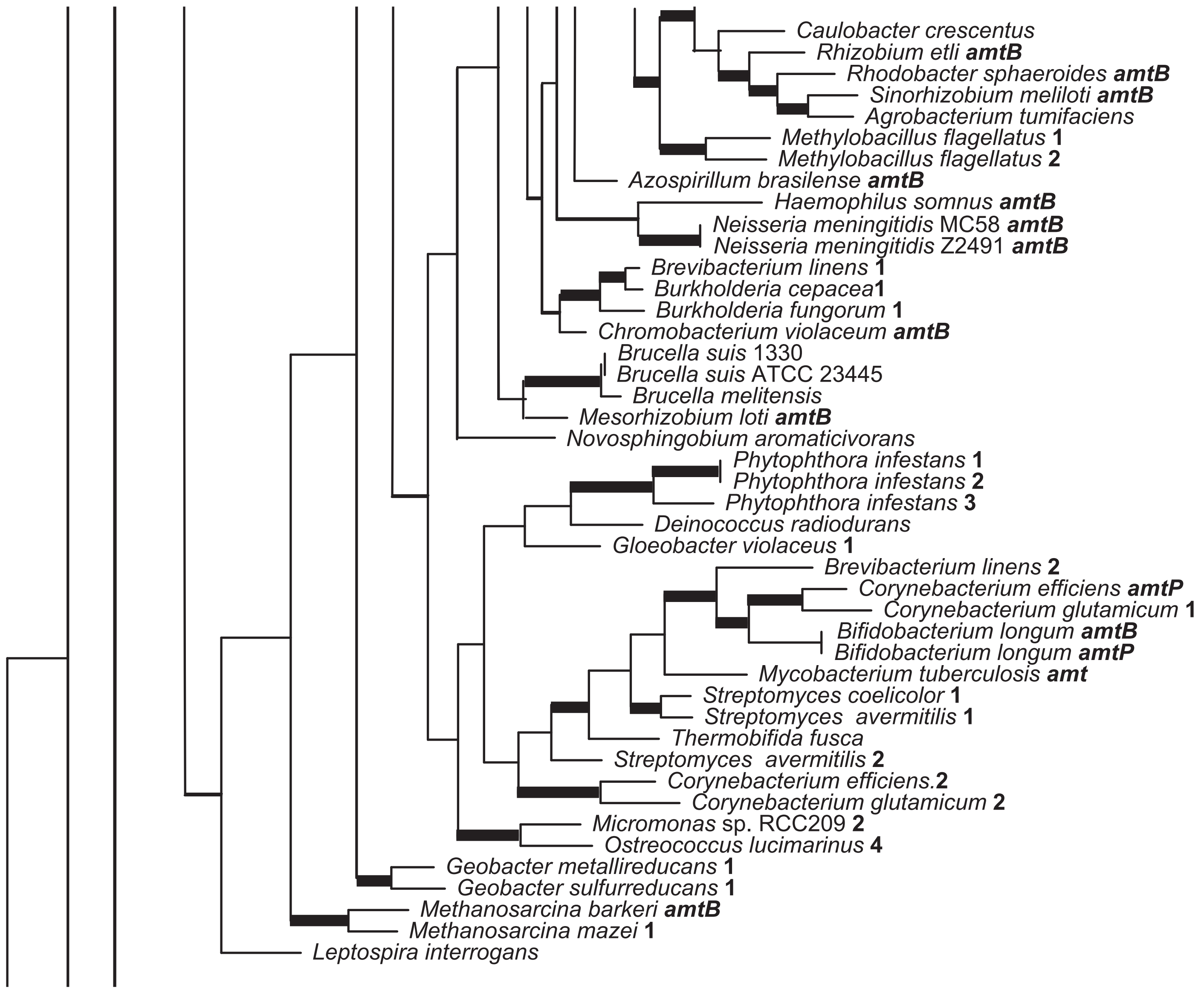
Caulobacter crescentus
Rhizobium etli amtB
Rhodobacter sphaeroides amtB
Sinorhizobium meliloti amtB
Agrobacterium tumifaciens
Methylobacillus flagellatus 1
Methylobacillus flagellatus 2
Azospirillum brasilense amtB
Haemophilus somnus amtB
Neisseria meningitidis MC58 amtB
Neisseria meningitidis Z2491 amtB
Brevibacterium linens 1
Burkholderia cepacea1
Burkholderia fungorum 1
Chromobacterium violaceum amtB
Brucella suis 1330
Brucella suis ATCC 23445
Brucella melitensis
Mesorhizobium loti amtB
Novosphingobium aromaticivorans
Phytophthora infestans 1
Phytophthora infestans 2
Phytophthora infestans 3
Deinococcus radiodurans
Gloeobacter violaceus 1
Brevibacterium linens 2
Corynebacterium efficiens amtP
Corynebacterium glutamicum 1
Bifidobacterium longum amtB
Bifidobacterium longum amtP
Mycobacterium tuberculosis amt
Streptomyces coelicolor 1
Streptomyces avermitilis 1
Thermobifida fusca
Streptomyces avermitilis 2
Corynebacterium efficiens.2
Corynebacterium glutamicum 2
Micromonas sp. RCC209 2
Ostreococcus lucimarinus 4
Geobacter metallireducans 1
Geobacter sulfurreducans 1
Methanosarcina barkeri amtB
Methanosarcina mazei 1
Leptospira interrogans

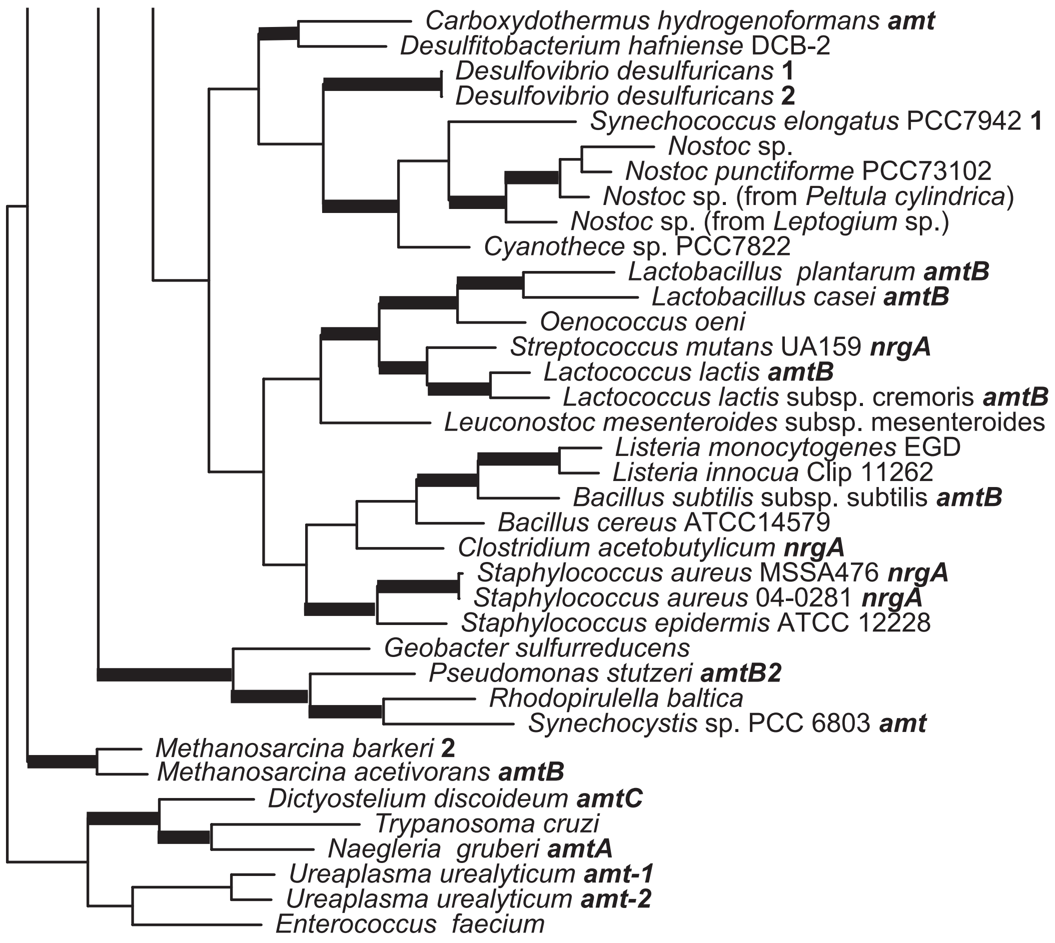

Carboxydothermus hydrogenoformans amt
Desulfitobacterium hafniense DCB-2
Desulfovibrio desulfuricans 1
Desulfovibrio desulfuricans 2
Synechococcus elongatus PCC7942 1
Nostoc sp.
Nostoc punctiforme PCC73102
Nostoc sp. (from Peltula cylindrica)
Nostoc sp. (from Leptogium sp.)
Cyanothece sp. PCC7822
Lactobacillus plantarum amtB
Lactobacillus casei amtB
Oenococcus oeni
Streptococcus mutans UA159 nrgA
Lactococcus lactis amtB
Lactococcus lactis subsp. cremoris amtB
Leuconostoc mesenteroides subsp. mesenteroides
Listeria monocytogenes EGD
Listeria innocua Clip 11262
Bacillus subtilis subsp. subtilis amtB
Bacillus cereus ATCC14579
Clostridium acetobutylicum nrgA
Staphylococcus aureus MSSA476 nrgA
Staphylococcus aureus 04-0281 nrgA
Staphylococcus epidermis ATCC 12228
Geobacter sulfurreducens
Pseudomonas stutzeri amtB2
Rhodopirulella baltica
Synechocystis sp. PCC 6803 amt
Methanosarcina barkeri 2
Methanosarcina acetivorans amtB
Dictyostelium discoideum amtC
Trypanosoma cruzi
Naegleria gruberi amtA
Ureaplasma urealyticum amt-1
Ureaplasma urealyticum amt-2
Enterococcus faecium
